# Hydrogen Sulfide, Ethylene, and Nitric Oxide Regulate Redox Homeostasis and Protect Photosynthetic Metabolism under High Temperature Stress in Rice Plants

**DOI:** 10.3390/antiox11081478

**Published:** 2022-07-28

**Authors:** Harsha Gautam, Mehar Fatma, Zebus Sehar, Iqbal R. Mir, Nafees A. Khan

**Affiliations:** Plant Physiology and Biochemistry Laboratory, Department of Botany, Aligarh Muslim University, Aligarh 202002, India; harshagautam99@gmail.com (H.G.); meharfatma30@gmail.com (M.F.); seharzebus5779@gmail.com (Z.S.); m3riqbal@gmail.com (I.R.M.)

**Keywords:** ethylene, hydrogen sulfide, nitric oxide, photosynthesis, rice

## Abstract

Rising temperatures worldwide due to global climate change are a major scientific issue at present. The present study reports the effects of gaseous signaling molecules, ethylene (200 µL L^−1^; 2-chloroethylphosphonic acid; ethephon, Eth), nitric oxide (NO; 100 µM sodium nitroprusside; SNP), and hydrogen sulfide (H_2_S; 200 µM sodium hydrosulfide, NaHS) in high temperature stress (HS) tolerance, and whether or not H_2_S contributes to ethylene or NO-induced thermo-tolerance and photosynthetic protection in rice (*Oryza sativa* L.) cultivars, i.e., Taipei-309, and Rasi. Plants exposed to an HS of 40 °C for six h per day for 15 days caused a reduction in rice biomass, associated with decreased photosynthesis and leaf water status. High temperature stress increased oxidative stress by increasing the content of hydrogen peroxide (H_2_O_2_) and thiobarbituric acid reactive substance (TBARS) in rice leaves. These signaling molecules increased biomass, leaf water status, osmolytes, antioxidants, and photosynthesis of plants under non-stress and high temperature stress. However, the effect was more conspicuous with ethylene than NO and H_2_S. The application of H_2_S scavenger hypotaurine (HT) reversed the effect of ethylene or NO on photosynthesis under HS. This supports the findings that the ameliorating effects of Eth or SNP involved H_2_S. Thus, the presence of H_2_S with ethylene or NO can enhance thermo-tolerance while also protecting plant photosynthesis.

## 1. Introduction

High temperature stress (HS) is significant environmental stress that restricts growth, metabolism, and crop production worldwide. Numerous biochemical processes susceptible to heat stress are involved in the growth and development of plants. Heat stress leads to the excess production of reactive oxygen species (ROS) that alter the cellular membrane protein structure and functions. Heat stress alters the expression of genes involved in direct protection from heat stress at the molecular level [1,2,3]. Crop production is currently a major concern due to HS, and methods for maintaining high crop yields under heat stress are prime agricultural objectives. Rice, a cereal crop belonging to the Poaceae family, is consumed by the majority of the world’s population. It can provide high productivity and a prominent position in the international rice trade of food grains [4]. Rice crops are frequently subjected to HS, which impacts their quality and use around the world. In defense, plants respond to heat stress in several ways, including accumulating solutes that can arrange proteins and cellular structures, maintaining cell turgor by osmotic adjustment, modifying the antioxidant system to re-establish cellular redox balance and homeostasis, and involving complex regulatory signaling molecules for protection from oxidative stress [3,5,6,7]. Phytohormones, as signaling molecules, are the crucial molecules for coordinating a wide range of plant growth and development processes. They are important as endogenous signaling molecules that play a role in mediating various physiological reactions under heat stress by activating stress-responsive regulatory genes involved in plant growth [3,8,9].

Ethylene, nitric oxide (NO), and hydrogen sulfide (H_2_S) have been identified as essential gaseous signaling molecules in plants and have recently attracted attention due to their participation in a number of physiological, biochemical, and cellular processes [4,7,10,11,12]. Ethylene is a plant hormone that regulates abiotic stress responses [13]. The treatment of ethephon (Eth; ethylene source) activates various stress-related proteins to preserve plant cell functional integrity and stability under heat stress [14]. Ethylene accumulation at varying concentrations is linked to plant responses to heat stress challenges that affect growth and development [15,16]. Ethylene signaling also enhances thermo-tolerance in plants by maintaining chlorophyll content and mitigating heat stress-induced adversity by reducing oxidative stress [17]. Recently, it was suggested that ethylene reduced glucose sensitivity and induced glutathione production, resulting in the increased expression of *psbA* and *psbB* genes to protect the pigment system (PS) II and photosynthesis under salt stress [9].

Nitric oxide has also emerged as a signaling molecule that masks the adverse effects of abiotic stresses such as heat, drought, salinity, ultraviolet (UV) radiation, heavy metals, etc., and received attention from the plant science community [7,18,19,20,21]. According to evidence, it appears to be a major signaling molecule in modulating various plant responses under heat stress, including photosynthesis, oxidative defense, gene expression, and protein changes. Nitric oxide modulates the heat stress transcription factors and DNA binding activity and acts upstream of AtCaM3 in heat stress signaling [22]. Nitric oxide plays a protective effect in PS II recovery in *Festuca arundinacea* under heat stress [18]. It scavenges ROS in plants [4,23] and increases the gene expression of *psbA* in maize [24], while CP43 and CP47 decrease under heat stress in rice [25]. Nitric oxide also upregulated the activities and expression of SOD, CAT, and APX genes in chickpea plants and mitigated the adverse effect of high salinity [26]. The consequences of NO’s regulation and the genetic and molecular evidence for its function in improving heat and cold stress tolerance and adaptation have led to the discovery of potential new techniques to deal with future environmental difficulties [27]. These studies emphasized the protective roles of NO against heat stress-induced direct damage to the crops.

Few recent studies emphasize the role of H_2_S with a diverse range of functions similar to NO in plants involved in various growths and development processes [7,21,28]. Recent research has linked H_2_S, an endogenously-produced signaling molecule, to the regulation of autophagy in both plants and mammals by persulfidating particular targets [29]. The close proximity of two modifications—persulfidation and phosphorylation—could influence one another and serve as integration points for the H_2_S- and ABA-signaling pathways [30]. Depending on the concentrations, both signaling molecules, NO and H_2_S, work synergistically or antagonistically in plants. The gap between NO and H_2_S is rapidly closing, and H_2_S is emerging as a critical signal mediator involved in various biological processes, including the modulation of multiple stress responses [31]. However, the function of NO and H_2_S in photosynthetic recovery processes during heat stress is still ambiguous. Hydrogen sulfide protects the crops and is involved in various physiological processes such as seed germination, root growth, stomatal movement, leaf wilting, fruit ripening, etc., under adverse environmental stress [11,28,32]. Additionally, H_2_S protects plants from heavy metals, salinity, drought, and extreme temperature stresses [33,34]. The study of Li et al. [35] suggested that H_2_S alleviated alkaline salt stress by regulating the expression of micro-RNAs through changes in the root architecture of *Malus hupehensis*. Hydrogen sulfide was influential in the thermo-tolerance in plants, and sodium hydrosulfide (NaHS)-pretreated seedlings (a H_2_S donor) decreased oxidative stress by increasing the action and gene expression of antioxidant enzymes, as well as soluble sugar levels in wheat [36]. Melatonin and H_2_S work together to protect against heat stress-induced photosynthetic inhibition by regulating carbohydrate metabolism, according to a study [11]. A few studies have also shown interactions between ethylene and H_2_S. A report observed that endogenous H_2_S is required for ethylene-mediated hexavalent chromium stress reduction in two pulse crops [37]. Similarly crucial for ethylene-induced stomatal closure in response to osmotic stress is ethylene-induced H_2_S, which is a downstream component of osmotic stress signaling [38]. In *Solanum lycopersicum*, ethylene and H_2_S co-treatment increased the expression of antioxidant-encoding genes *SlAPX2*, *SlCAT1*, *SlPOD12*, and *SlCuZnSOD* compared to ethylene treatment alone [39].

Thus, it was hypothesized that under HS, ethylene, NO, and H_2_S may play a critical role in plant defense and cause considerable improvements in thermo-tolerance in plants by influencing multiple pathways. However, until now, there is no study available that correlates the study of ethylene, NO, and H_2_S under HS. Thus, the present study highlights the impact of HS on ethylene, NO, and H_2_S-mediated mechanisms and traits associated with thermo-tolerance and the involvement of H_2_S in ethylene or NO-induced management strategies for oxidative stress-signaling and defense systems in rice plants.

## 2. Materials and Methods

### 2.1. Plant Material, Growth Conditions, and Experimental Design

Rice (*Oryza sativa* L.) cultivars, Taipei-309 (HS-tolerant) and Rasi (HS-non tolerant), obtained from the Indian Agricultural Research Institute, New Delhi were selected for the study. Their tolerance to HS was determined after their screening for changes in photosynthesis, growth, and yield parameters relative to controls, according to our earlier work [4]. After sterilizing the seeds with HgCl_2_ (0.01%) for 2 min and rinsing repetitively with double distilled water, they were soaked in distilled water for 12–24 h and then incubated at 30 °C. Following incubation, the seeds were sown in 23 cm diameter pre-sterilized earthen pots containing 4 kg of acid-washed sand. Ten seeds of each cultivar were initially sown per pot, and later three seedlings were maintained after thinning. The pots were placed in an environmental growth chamber (Khera Instruments, New Delhi, India) with a day/night regime of 16/8 h, photosynthetically active photon flux density (PPFD) of 200 µmol m^−2^ s^−1^ at plant level, the temperature of 28 °C in the light and 22 °C in the dark, and relative humidity of 65 ± 5%.

In the experimentation, the plants were subjected to HS by exposing them to 40 °C temperature for six h daily, and the heat treatment was administered ten days after sowing (DAS). The heat treatment was maintained for 15 days for the same duration. After that, the plants were allowed to grow for five days at 28 °C (optimum temperature). The experimentation continued for 30 days. Control plants were kept at 28 °C for the duration of the experiment (30 days). A concentration of 200 µL L^−1^ 2-chloroethyl phosphonic acid (Eth; as an ethylene donor), 100 µM sodium nitroprusside (SNP; as a NO donor), and 200 µM NaHS was applied to the foliage of both HS-treated and non-treated plants with a hand sprayer at 15 DAS. Moreover, 100 µM hypotaurine (HT; H_2_S scavenger), 100 µM norbornadiene (NBD; ethylene action inhibitor) and 100 µM 2-4-carboxyphenyl-4,4,5,5 -tetramethylimidazoline-1-oxyl-3-oxide (cPTIO; NO scavenger) were also applied to heat-stressed plants. The concentration of 100 µM cPTIO [7], 100 µM NBD [40], and 100 µM HT [11] used was based on our earlier studies. A surfactant teepol (0.5%) was added with the control and other treatment solutions. Our experimental design consisted of twelve treatments as follows: (i) control, (ii) HS, (iii) Eth, (iv) SNP, (v) NaHS, (vi) HS + Eth, (vii) HS + SNP, (viii) HS + NaHS, (ix) HS + Eth + HT, (x) HS + SNP + HT, (xi) HS + NaHS + NBD, and (xii) HS + NaHS + cPTIO. The hydrolysis of ethephon releases ethylene and phosphate, and the yield of phosphate is equivalent to ethylene [41,42]. Thus, the phosphate effect was nullified by adjusting the phosphate available from 200 µL L^−1^ Eth as single super phosphate. The arrangement of the treatments was a complete randomized block design with four replicates for each treatment (*n* = 4). The sampling of the plants was performed at 30 DAS to record various parameters of interest.

### 2.2. Measurement of Photosynthetic and Growth Characteristics

The Infrared Gas Analyzer (CID-340, Photosynthesis System, Bio-science, Washington, WI, USA) was used to measure gas exchange photosynthetic parameters (net photosynthetic rate, stomatal conductance, and intercellular CO_2_ concentration) in the fully expanded upper leaves. At the time of measurements (between 11.00 and 12.00 h), the atmospheric CO_2_ concentration was 380 ± 5 µmol mol^−1^, the relative humidity was 70%, the photosynthetic active radiation was 780 µmol m^−2^ s^−1^, and the air temperature was 28 °C. The chlorophyll content was measured in intact upper leaves of the plants with a SPAD chlorophyll meter (SPAD 502 DL PLUS, Spectrum Technologies, Plainfield, IL, USA) in the early morning hours. The dry weight of shoots and roots was recorded after separating the plants into roots and shoots, washed with water, and blotted with a soft paper towel to remove excess moisture. The separated shoots and roots were dried in a hot air oven (80 °C) for 72 h until a constant weight was achieved.

### 2.3. Chlorophyll Fluorescence Measurement

Fully expanded leaves were allowed to adapt under dark for 30 min before chlorophyll fluorescence measurements using Junior-PAM chlorophyll fluorometer (Heinz Walz GmbH, Eichenring, Effeltrich, Germany) were taken. The actual efficiency of PS II (ΦPSII), maximal efficiency of PS II (Fv/Fm), intrinsic efficiency of PS II (Φesc), photochemical quenching (qP), non-photochemical quenching (NPQ), and electron transport rate (ETR) were calculated. The details of the procedure are given in Supplementary File S1.

### 2.4. Leaf Relative Water Content (RWC) Determination

Leaf RWC was measured following the method of Barrs and Weatherley [43]. Fresh leaves were collected, weighed instantly by a standardized weighing balance, and then dipped into water in separate Petri-dishes for 12 h. The turgid weight was calculated by weighing the wet leaves. Afterwards, the leaf samples were oven-dried at 80 °C for 48 h, and the dry weight was recorded. The RWC was calculated using the following formula:

RWC (%) = [(Fresh weight − Dry weight)/(Turgid weight − Dry weight)] × 100.

### 2.5. Estimation of the Contents of Proline, Glycine Betaine (GB), Trehalose and Soluble Sugars

Proline content was determined by adopting the ninhydrin method [44]. Briefly, fresh leaf tissues (300 mg) were homogenized in 3 mL of 3% sulphosalicylic acid, and the homogenate filtrate was reacted with 1 mL each of acid ninhydrin and glacial acetic acid for 1 h in a test tube placed in a water bath at 100 °C. The mixture was extracted with toluene, and the absorbance was measured on a spectrophotometer at 520 nm using L-proline as a standard.

Glycine betaine was determined by estimating the betaine-periodite complex [45] in a sample from 500 mg dried leaf powder mechanically shaken for 24 h at 25 °C with 20 mL of deionized water. After filtering the samples, the filtrates were diluted (1:1) with 2 N H_2_SO_4_. A portion (0.5 mL) was taken and centrifuged before cooling in ice water for one hour. After adding 0.2 mL of cold KI-I_2_ reagent, the reactants were gently stirred. The tubes were kept at 4 °C for 16 h and were centrifuged at 10,000× *g* for 15 min at 0 °C. After carefully aspirating the supernatant, the absorbance at 365 nm was measured after two hours. In 2 N H_2_SO_4_, reference standards for GB (50–200 µg mL^−1^) were created. The trehalose content was determined following the protocol given by Trevelyan and Harrison [46]. Dried leaf powder (500 mg) was extracted in 80% ethanol, followed by centrifugation at 5000× *g* for 15 min at 4 °C. A 100 µL sample of the supernatant was combined with 4 mL of anthrone reagent and 2.0 mL of trichloroacetic acid (TCA). The absorbance was read at 620 nm. A standard curve was plotted using glucose.

The method developed by Xu et al. [47] was used to measure the amount of soluble sugars. A total of 100 mg of the dried sample powder was extracted with 10 mL of 80% ethanol and incubated at 80–85 °C for 30 min. Three additional extractions were performed after centrifuging the extract and transferring the supernatant to a 100 mL volumetric flask. At 80–85 °C, alcohol extract was evaporated over a water bath. Following the addition of 100 mL distilled water, all three supernatants were poured into the flask. An aliquot of the extract was used to measure the amount of soluble sugars using the anthrone reagent, and the reaction mixture’s absorbance was observed at 630 nm using a spectrophotometer.

### 2.6. Measurement of Hydrogen Peroxide (H_2_O_2_) and Lipid Peroxidation

The Okuda et al. [48] method was used for the H_2_O_2_ assay and is explained earlier [49]. The details of the method are given in Supplementary File S1. Fresh leaf tissues (500 mg) were macerated in ice-cold 200 mM perchloric acid before being spun at 1200× *g* for 10 min. Later, 4 M KOH was used to neutralize the supernatant. In order to remove the insoluble potassium perchlorate from the homogenate, it was further centrifuged at 500× *g* for three minutes. The reaction mixture (1.5 mL) included 20 µL of peroxidase (0.25 unit), 400 µL of 12.5 mM 3-(dimethylamino) benzoic acid in 0.375 M phosphate buffer (pH 6.5), 80 µL of 3-methyl-2-benzothiazoline hydrazone, and 1 mL of the eluate. Peroxidase was added and the reaction was started at 25 °C. On a spectrophotometer, the increase in absorbance was calculated at 590 nm. The content of thiobarbituric acid reactive substances (TBARS) was estimated to determine lipid peroxidation as described by Dhindsa et al. [50] and explained earlier [49].

### 2.7. Assay of Antioxidant Enzymes Activities

The activity of superoxide dismutase (SOD), ascorbate peroxidase (APX), and glutathione reductase (GR) was measured using the methods of Beyer and Fridovich [51], Giannopolitis and Ries [52], Nakano and Asada [53], and Foyer and Halliwell [54], respectively. The details of the methods are given in Supplementary File S1.

### 2.8. Determination of Nitric Oxide, Hydrogen Sulfide, and Ethylene 

The method of Zhou et al. [55] was used for determining NO content with a slight modification by estimating nitrite content. Using a pre-chilled mortar and pestle, 500 mg of healthy leaves were ground in 3.0 mL of 50 mM ice-cold acetic acid buffer (pH 3.6) containing 4% zinc acetate. The mixture was then centrifuged at 11,500× *g* for 15 min at 4 °C. The supernatant was collected, and the pellets were rinsed in extraction buffer (1.0 mL) before being centrifuged again. After adding 100 mg of charcoal, the supernatants from the two spins were neutralized. The filtrate was collected after a brief vortex. Greiss reagent (0.1% N-1-naphthyl ethylenediamine dihydrochloride and 1% sulphanilamide in 5% H_2_PO_4_ solution) was added to each 1.0 mL filtrate and mixed in a 1:1 ratio before incubation at room temperature for 30 min. The NO content was determined using a calibration curve with sodium nitrite as a standard, and the absorbance was measured at 540 nm. Methylene blue formation from dimethyl-p-phenylenediamine in HCl was used to estimate the content of leaf H_2_S as described by Xie et al. [56] with minor modifications. The fresh leaf samples (700 mg) were homogenized in 2.5 mL of Tris-HCl buffer (20 mM L^−1^, pH 6.8) containing 10 mM L^−1^ ethylene diamine tetraacetic acid (EDTA). The homogenate was centrifuged for 15 min at 4 °C and 12,000× *g*. For H_2_S trapping, 0.2 mL of 1% (*w*/*v*) zinc acetate was added to the supernatant (0.75 mL). After 30 min of development, 0.1 mL of 30 mM L^−1^ ferric chloride in 1.2 mol L^−1^ of HCl and 0.1 mL of 20 mM L^−1^ dimethyl-p-phenylenediamine dissolved in 7.2 mol L^−1^ of HCl were added. At 670 nm, spectrophotometric analysis was used to determine the methylene blue formation. As a standard curve, different concentrations of NaHS were used, expressed as nmol g^−1^ fresh weight (FW).

The ethylene evolution in leaves was measured using a gas chromatograph following the procedure as described earlier by Fatma et al. [12]. The details are given in Supplementary File S1.

### 2.9. RNA Isolation and cDNA Synthesis

Following the manufacturer’s instructions, total RNA was extracted from rice leaves using the TRIzol reagent (Ambion, Life Technologies, Austin, TX, USA). With the help of a Nanodrop spectrophotometer (Thermo Scientific, Waltham, MA, USA), the extracted RNA was quantified. The details of the procedure are given in Gautam et al. [49] and presented in Supplementary File S1.

### 2.10. Quantitative Real-Time PCR Analysis

Real-time PCR (RT-PCR) was performed in a 96-well reaction plate (Roche, Mannheim, Germany) containing 20 µL reaction mixture of ×10 reaction buffer, 2 mM dNTPs, 1 mM MgCl_2_, 0.35 µM each of forward and reverse primers, 1 µL Sybr green (×10), 10 µg cDNA template, and 5 U Taq polymerase on a thermal cycler (Light cycler 480 II, Roche, Germany). The details of the procedure are given in Gautam et al. [49] and presented in Supplementary File S1. Primer pairs used for the quantitative RT-PCR are listed in Supplementary Table S1. The data was interpreted as the differential expression of the target gene in the treated sample versus the untreated control in relation to the internal control.

### 2.11. Statistical Analysis

A two-way analysis of variance (ANOVA) was performed with SPSS software version 17.0 for Windows to analyze the data and presented as a treatment mean ± SE (*n* = 4). The least significant difference (LSD) was calculated for the significant data at *p* < 0.05. The data bars with the same letter were not significantly different by the LSD test at *p* < 0.05. The principal component analysis (PCA) and Pearson correlation analyses ((*p* < 0.05, *p* < 0.01 and *p* < 0.001) were carried out using OriginPro software. To create biplots, the first two components (PC1 and PC2) showing the maximum variance in the datasets were considered.

## 3. Results

### 3.1. Growth Parameters

**Figure 1:**
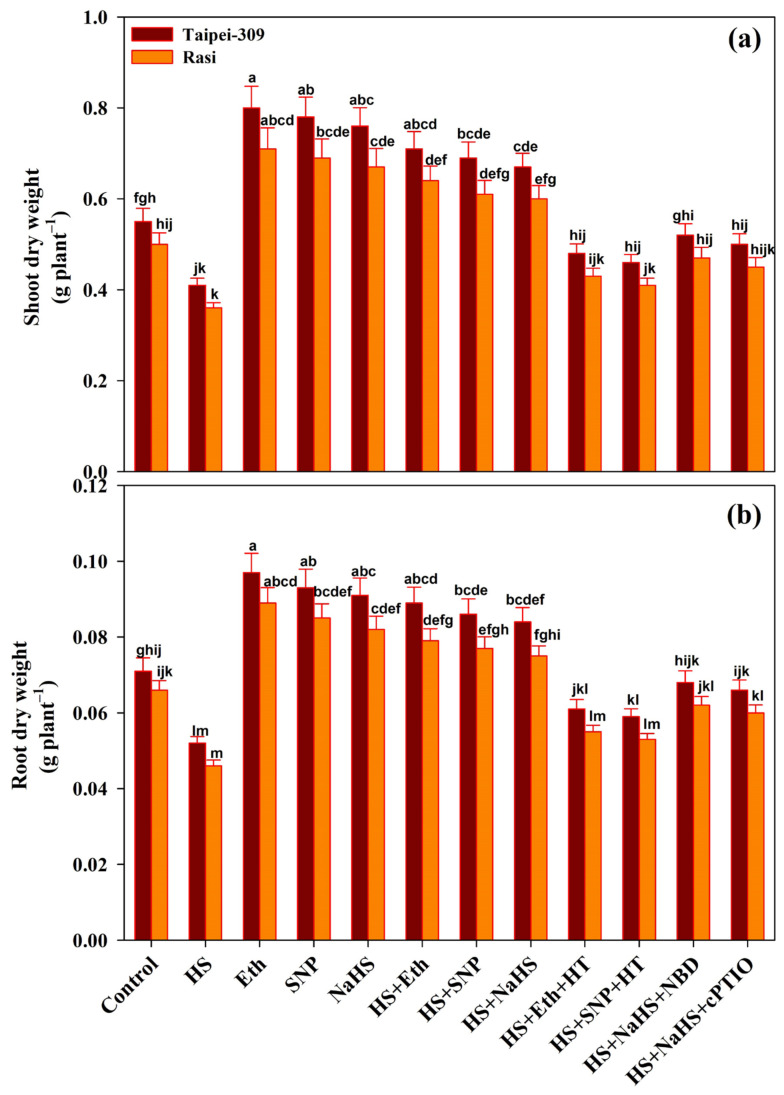
(**a**) Shoot dry weight and (**b**) root dry weight of rice (*Oryza sativa* L.) cultivars Taipei-309 and Rasi under control and high temperature stress (HS) supplied with 200 µL L^−1^ ethephon (Eth), 100 µM sodium nitroprusside (SNP), 200 µM sodium hydrosulfide (NaHS) or 100 µM hypotaurine (HT), 100 µM 2-4-carboxyphenyl-4,4,5,5 -tetramethylimidazoline-1-oxyl-3-oxide (cPTIO) or 100 µM norbornadiene (NBD) scavengers of hydrogen sulfide (H_2_S), nitric oxide (NO), and ethylene action inhibitors, respectively. Data are presented as treatments mean ± SE (*n* = 4). The values followed by the same letters did not differ significantly by LSD test at *p* < 0.05.

The rice plants’ growth was analyzed in terms of shoot and root dry weight. The plants subjected to HS showed a reduction in shoot dry weight by (25.4%) in Taipei-309 and (28.0%) in Rasi, and root dry weight by (26.7%) in Taipei-309 and (30.3%) in Rasi, in comparison to control. However, Eth, SNP or NaHS application resulted in the reversal of the adverse effects of HS on rice growth; as the shoot dry weight recovered by Eth (29.0% and 28.0%), SNP (25.4% and 22.0%), and NaHS (21.8% and 20.0%) in Taipei-309 and Rasi, respectively, compared to control plants (Figure 1).

In Taipei-309 and Rasi, foliar applications of Eth, SNP, or NaHS lowered the effect of HS on root dry weight by (25.3% and 19.6%), (21.1% and 16.6%), or (18.3% and 13.6%), respectively, compared to controls.

This suggests that Eth, SNP, or NaHS can reduce the negative effects of HS on the biomass of both rice cultivars. Moreover, the application of HT exacerbated the deleterious effects of HS in Taipei and Rasi cultivars, resulting in reduced shoot dry weight by (12.7% and 14.0%) and by (16.3% and 18.0%) in the presence of Eth and SNP, respectively. Similarly, the root dry weight was reduced by (14.0% and 16.6%) and by (16.9% and 19.6%) in Eth and SNP-treated Taipei-309 and Rasi, respectively, relative to control plants. Therefore, combining HT with Eth or SNP completely reversed the mitigating effects of Eth or SNP. The application of NBD and cPTIO reduced shoot and root dry weights in the presence of NaHS in heat-treated plants in both the cultivars, reflecting that the application of NBD or cPTIO with NaHS did not entirely reverse the mitigating effects of NaHS on growth characteristics.

### 3.2. Gas-Exchange Parameters and Chlorophyll Content

Net photosynthesis rate (Pn), stomatal conductance (Gs), intercellular CO_2_ concentration (Ci), and SPAD value decreased under HS by 35.0%, 26.9%, 23.2%, and 28.4% in Taipei-309 and 36.1%, 28.2%, 24.2%, and 29.2% in Rasi, respectively, compared to control plants (Table 1). In comparison to control and heat-stressed plants, the individual treatment of Eth, SNP, and NaHS increased these parameters significantly. The individual applications of Eth, SNP, or NaHS under HS showed a substantial increase in *Pn* (29.1% and 98.9%, 27.1% and 95.9%, 24.5% and 91.8%), *Gs* (25.6% and 71.9%, 25.0% and 71.8%, 24.4% and 70.3%), *Ci* (21.3% and 57.9%, 20.2% and 56.6%, 19.9% and 56.1%), and SPAD value (37% and 91.7% and 35.9% and 90%, 32% and 84.6%) in Taipei-309 and *Pn* (25.6% and 96.7%, 24.3% and 94.5%, 20.8% and 89%), *Gs* (23.1% and 71.6%, 21.9% and 70%, 21.4% and 69.3%), *Ci* (18.7% and 56.8%, 17.8% and 55.6%, 16.2% and 53.4%), and SPAD value (35.8% and 91.9%, 31.4% and 85.6%, 27.6% and 80.2%) in Rasi, respectively. Exogenously-applied Eth considerably alleviated more compared to SNP or NaHS, the decrement in the levels of *Pn*, *Gs*, *Ci*, and SPAD caused by HS. However, the potential effects of Eth or SNP on these parameters were significantly minimized by the H_2_S scavenger, HT. The treatment of NBD or cPTIO moderately reversed the mitigating effects of NaHS.

### 3.3. Chlorophyll Fluorescence Parameters

The measurement of chlorophyll fluorescence parameters was taken under both stress and without stress in leaves of rice cultivars (Table 2 and Table 3). Heat stress exposure reduced the studied fluorescence parameters compared to control, but treatment with Eth, SNP, or NaHS increased these parameters under stress and no stress conditions. In contrast, NPQ increased under HS but decreased significantly with Eth, SNP, or NaHS application under no stress compared to control. Eth, SNP or NaHS treatments proved effective in improving (ΦPS II, Fv/Fm, Φesc, qP, and ETR) in heat-treated plants compared to controls. The data revealed that Eth, SNP, or NaHS treatments were essential to mitigate the negative effects of HS on the parameters mentioned above. Still, Eth was more effective than SNP or NaHS. The application of NBD or cPTIO reversed the positive effects of NaHS on the chlorophyll fluorescence parameters under HS; however, the addition of HT (100 µM) along with Eth or SNP entirely reversed the positive effects of Eth or SNP on these parameters in heat-stressed conditions.

### 3.4. Leaf RWC

**Figure 2:**
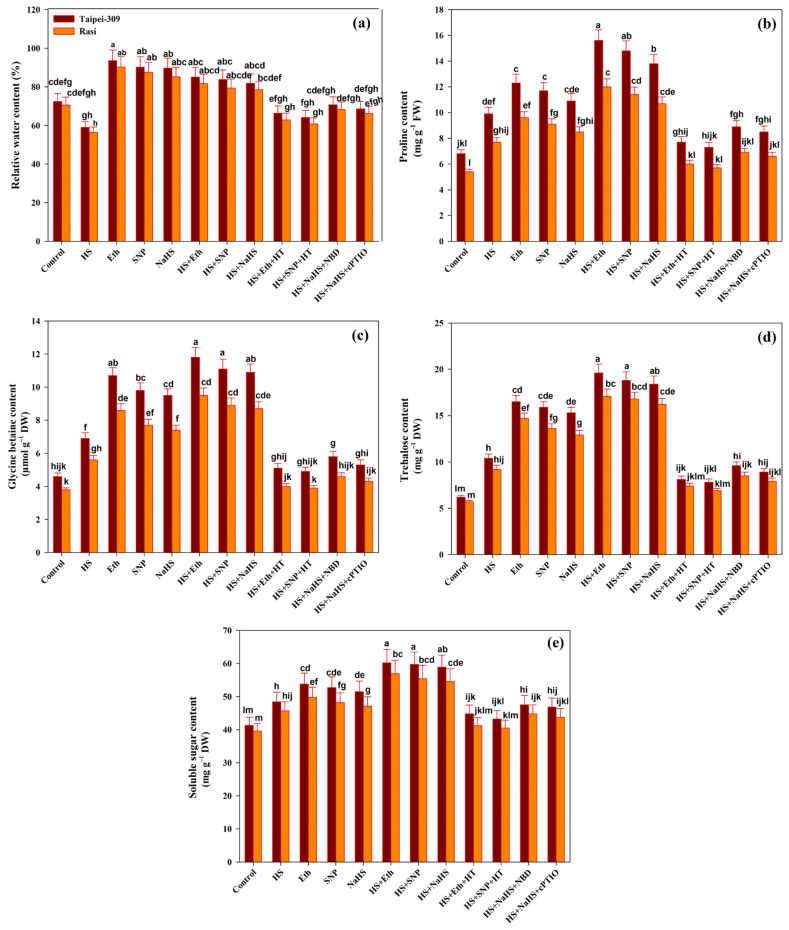
(**a**) Relative water content, (**b**) proline content, (**c**) glycine betaine content, (**d**) trehalose content, and (**e**) soluble sugars content of rice (*Oryza sativa* L.) cultivars Taipei-309 and Rasi under control and high temperature stress (HS) supplied with 200 µL L^−1^ ethephon (Eth), 100 µM sodium nitroprusside (SNP), 200 µM sodium hydrosulfide (NaHS), or 100 µM hypotaurine (HT), 100 µM 2-4-carboxyphenyl-4,4,5,5 -tetramethylimidazoline-1-oxyl-3-oxide (cPTIO) or 100 µM norbornadiene (NBD) scavengers of hydrogen sulfide (H_2_S), nitric oxide (NO), and ethylene action inhibitors, respectively. Data are presented as treatments mean ± SE (*n* = 4). The values followed by the same letters did not differ significantly by LSD test at *p* < 0.05.

The HS decreased the leaf RWC of both the cultivars by (18.5%) in Taipei-309 and (20.1%) in Rasi compared to control plants (Figure 2a). The results revealed that Eth-, SNP-, or NaHS-spraying treatments improved the RWC in no stress and HS conditions. In heat-stressed plants, Eth recovered RWC by (17.5% and 44.2%), SNP by (15.7% and 42%) or NaHS by (13.1% and 38.8%) in Taipei-309; and by (15.7% and 44.8%), (12.4% and 40.7%) or by (11.1% and 39.1%) in Rasi compared to control and heat-stressed plants, respectively. Overall, maximum RWC was recorded in plants treated with Eth, followed by SNP or NaHS spraying treatments under control and heat-stressed conditions. Furthermore, in heat-exposed plants, the combined application of (NBD and NaHS) and (cPTIO and NaHS) reduced RWC, relative to control plants. The addition of HT along with Eth or SNP under HS more drastically declined RWC, reversing the beneficial effects of Eth or SNP treatments on RWC.

### 3.5. Accumulation of Osmolytes

Plants accumulate osmolytes or compatible solutes to protect the cellular machinery from various environmental stresses. Glycine betaine, sugars (trehalose), and proline are the most well-known osmolytes. The treatment of HS increased the proline and GB content by 45.5% and 50% in Taipei-309 and 42.5% and 47.3% in Rasi, respectively, compared to control plants (Figure 2b,c). With Eth, SNP, or NaHS application under no stress, the levels of proline and GB increased appreciably. The application of Eth improved proline content (129.4% and 57.5%) in Taipei-309 and (122.2% and 55.8%) in Rasi, and GB content (156.5% and 71.0%) in Taipei-309 and (150% and 69.6%) in Rasi, respectively, under stressful condition compared to control and heat-stressed plants.

Improvements in proline and GB contents were also observed by SNP application under HS conditions (117.6% and 111.1%) in Taipei-309 and (141.3% and 134.2%) in Rasi, respectively, compared to control plants. Foliar-applied NaHS enhanced proline content (102.9% and 98.1%) and GB content (136.9% and 128.9%) in Taipei-309 and Rasi, respectively, compared to controls. Under HS stress, the treatment of HT completely suppressed the beneficial effects of Eth or SNP on proline and GB accumulation. At the same time, NBD or cPTIO could not considerably inhibit the effects induced by NaHS on proline and GB content in the leaves of heat-stressed plants. A considerable increase in trehalose and soluble sugar accumulation was recorded in heat-stressed plants of both rice cultivars, with Taipei-309 (67.7%) and (17.1%) with a higher accumulation than Rasi (61.4%) and (15.4%), respectively, compared to control plants (Figure 2d,e). Under no stress conditions, foliar treatment with Eth, SNP, or NaHS alone further increased the trehalose and soluble sugars content, although the increase was cultivar specific. The trehalose and soluble sugars content also increased considerably in both rice cultivars due to exogenously-applied Eth, SNP, or NaHS alone under HS.

Eth application increased trehalose and soluble sugars content (216.1% and 200.0%) and (45.7% and 43.6%) while SNP increased (203.2% and 194.7%) and (44.5% and 39.8%), and NaHS (196.7% and 184.2%) and (42.6% and 37.8%) in Taipei-309 and Rasi, respectively, under heat-stressed conditions, compared to control plants. However, even NBD and cPTIO did not completely inhibit NaHS from accumulating trehalose and soluble sugars. Furthermore, trehalose and soluble sugars accumulation by Eth and SNP was not sustained on the addition of HT to Eth and SNP-supplemented heat-stressed plants, which supported the role of H_2_S in ethylene and NO-induced osmolytes accumulation under HS.

### 3.6. Oxidative Stress

The oxidative stress was measured as H_2_O_2_ and TBARS. The plants subjected to HS showed a rise in the content of H_2_O_2_ by 3.0 fold in Taipei-309 and by 3.2 fold in Rasi, as well as TBARS content by 2.0 fold in Taipei-309 and by 2.1 fold in Rasi, compared to control plants (Table 4 and Table 5). However, the spraying of Eth, SNP, or NaHS reduced the contents of H_2_O_2_ and TBARS in both heat-stressed and non-stressed conditions. The Eth application reduced heat-induced oxidative stress, as evidenced by the observed reductions in the levels of H_2_O_2_ (66.0% and 67.5%) and TBARS (50.6% and 50.9%) in Taipei-309 and Rasi, respectively, compared to heat-stressed plants. Under HS, the application of SNP or NaHS decreased the contents of H_2_O_2_ by 65.1% and 64.3% in Taipei-309 and by 66.3% and 66.0% in Rasi, as well as TBARS content by 49.3% and 47.9% in Taipei-309 and by 49.0% and 47.1% in Rasi, respectively, relative to the values of heat-treated plants. These findings suggest that the individual treatment of Eth, SNP, or NaHS mitigated heat-induced oxidative stress by lowering the accumulation of H_2_O_2_ and TBARS. However, the addition of HT further stimulated H_2_O_2_ and TBARS in both rice cultivars under HS. Furthermore, Eth and SNP did not rescue the negative effects of HT on H_2_O_2_ and TBARS accumulation. Therefore, the application of HT completely reversed the alleviating effects of both Eth and SNP. The application of NBD or cPTIO reversed the reduced oxidative stress induced by NaHS.

### 3.7. Antioxidants Enzyme Activity

The activity of antioxidants, SOD, APX, and GR was studied to investigate the regulatory role of ethylene, NO, or H_2_S in the alleviation of HS-induced oxidative stress (Table 4 and Table 5). The increases in the activity of SOD (48.4% and 45.9%), APX (43.7% and 41.9%), and GR (42.1% and 31.2%) in Taipei-309 and Rasi, respectively, were noted under HS compared to control plants. Applying Eth to heat-treated plants showed a higher increase in SOD, APX, and GR activity than SNP compared to control or heat-stressed plants in both cultivars. Similarly, NaHS stimulated SOD, APX, and GR activity in both cultivars compared to control or heat-stressed plants.

The combined application of (NBD and NaHS) and (cPTIO and NaHS) in heat-treated plants did not entirely counteract the beneficial effect of NaHS on antioxidative enzyme activity. In addition, HT application in heat-stressed plants completely reversed the positive effects of Eth and SNP on the antioxidant defense system. Thus, HT supplementation resulted in reduced antioxidative enzyme activity in Eth and SNP-treated heat-stressed plants.

### 3.8. Hydrogen Sulfide and NO Content and Ethylene Production

**Figure 3:**
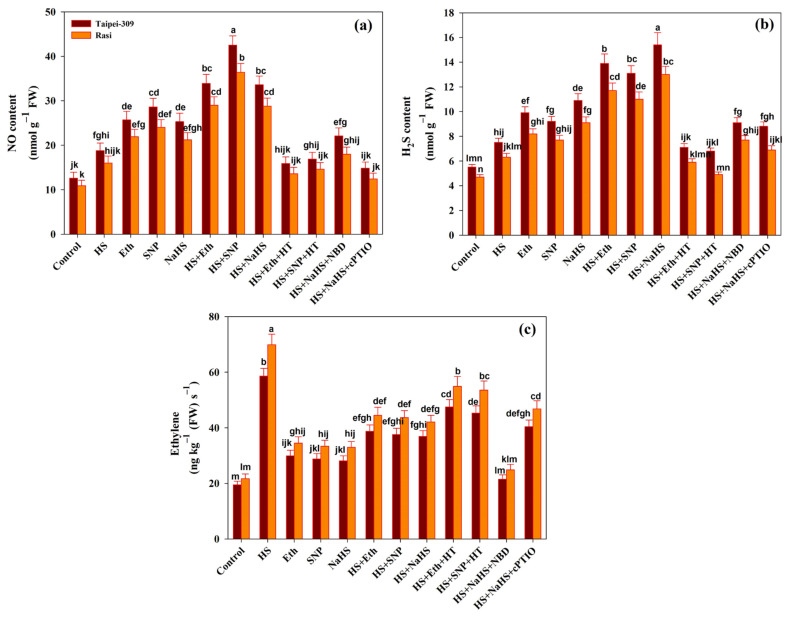
Content of (**a**) Nitric oxide (NO), (**b**) hydrogen sulfide (H_2_S), and (**c**) ethylene evolution in rice (*Oryza sativa* L.) cultivars Taipei-309 and Rasi under control and high temperature stress (HS) supplied with 200 µL L^−1^ ethephon (Eth), 100 µM sodium nitroprusside (SNP), 200 µM sodium hydrosulfide (NaHS) or 100 µM hypotaurine (HT), 100 µM 2-4-carboxyphenyl-4,4,5,5-tetramethylimidazoline-1-oxyl-3-oxide (cPTIO), or 100 µM norbornadiene (NBD) scavengers of hydrogen sulfide (H_2_S), NO, and ethylene action inhibitors, respectively. Data are presented as treatments mean ± SE (*n* = 4). The values followed by the same letters did not differ significantly by LSD test at *p* < 0.05.

The endogenous content of H_2_S and NO in rice cultivar leaves was examined to evaluate the effects of HS on H_2_S and NO regulation. Figure 3a,b depicts increased NO and H_2_S contents in the leaves of rice cultivar plants subjected to HS. The treatment of HS increased NO levels by 49.2% and 46.7% and H_2_S levels by 36.3% and 34.0% in Taipei-309 and Rasi, respectively, compared to control plants. Exogenously-applied Eth, SNP, or NaHS enhanced the level of both NO and H_2_S in rice cultivar leaves compared to control and heat-stressed plants. The treatment of HT and Eth or SNP under HS reduced endogenous NO and H_2_S levels relative to heat-treated plants. Similarly, applying cPTIO and NaHS under HS reduced NO levels compared to stressed and controlled plants.

The individual application of NBD and cPTIO and NaHS under HS did not affect H_2_S levels compared to control and heat-stressed plants.

Ethylene production in leaves of rice cultivars exposed to HS is depicted in Figure 3c. Compared to control plants, HS elevated ethylene levels by 200.5% and 222.1% in Taipei-309 and Rasi, respectively. Although SNP, NaHS, or Eth individually increased ethylene emission, the increase was less than that of plants that were subjected to high temperatures. Compared to heat-treated plants, plants that received Eth, SNP, or NaHS under stressful conditions showed a reduction in ethylene emission. The inhibition of H_2_S and NO using inhibitors HT and cPTIO, respectively, increased ethylene levels relative to control plants. Ethylene action inhibitor NBD application along with NaHS under HS decreased ethylene level compared to heat-stressed plants.

### 3.9. Expression of Photosynthesis-Related Genes and Genes Encoding Antioxidant Enzymes

**Figure 4:**
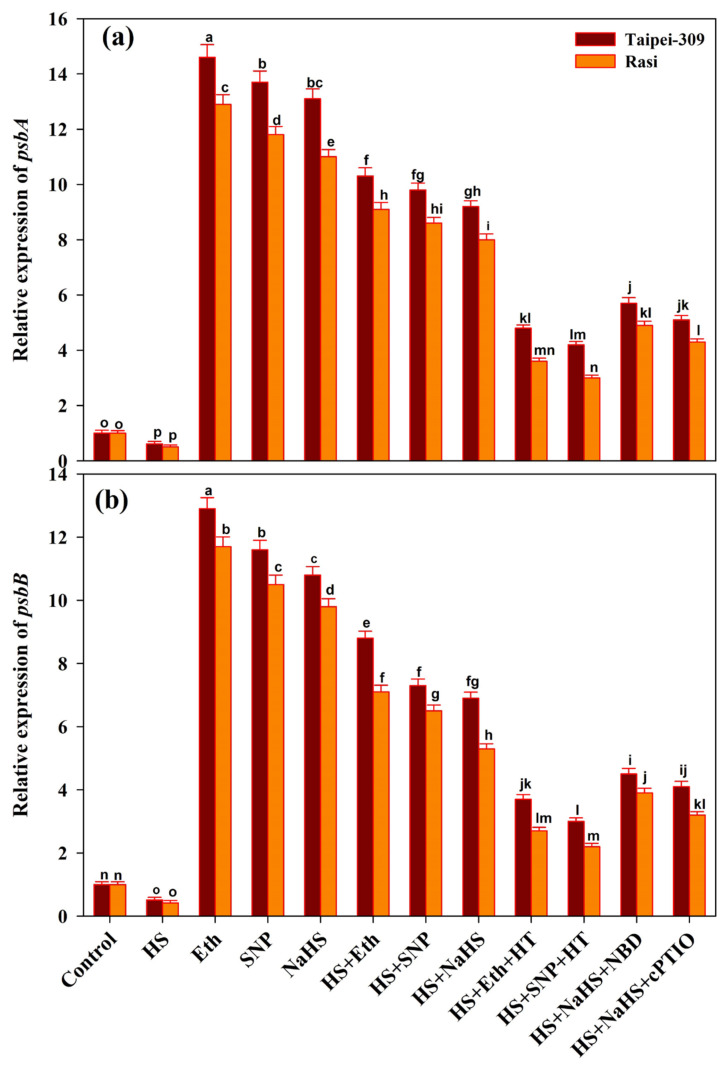
Relative expression of (**a**) *psbA* and (**b**) *psbB* of rice (*Oryza sativa* L.) cultivars Taipei-309 and Rasi under control and high temperature stress (HS) supplied with 200 µL L^−1^ ethephon (Eth), 100 µM sodium nitroprusside (SNP), 200 µM sodium hydrosulfide (NaHS) or 100 µM hypotaurine (HT), 100 µM 2-4-carboxyphenyl-4,4,5,5-tetramethylimidazoline-1-oxyl-3-oxide (cPTIO), or 100 µM norbornadiene (NBD) scavengers of hydrogen sulfide (H_2_S), nitric oxide (NO), and ethylene action inhibitors, respectively. Data are presented as treatments mean ± SE (*n* = 4). The values followed by the same letters did not differ significantly by LSD test at *p* < 0.05.

The expression of two genes relevant to the photosynthetic system was investigated in rice cultivars under HS (Figure 4a,b). The treatment of HS downregulated the expression of *psbA* and *psbB* in rice cultivar leaves, whereas heat-treated plants supplemented with Eth, SNP, or NaHS had higher levels of *psbA* and *psbB* transcription than control plants. In addition, under HS, exogenous HT with Eth or SNP significantly down-regulated the expression of *psbA* and *psbB* compared to the heat-treated Eth or SNP-supplemented plants. The expression of *psbA* and *psbB* was reduced in NBD or cPTIO with NaHS-treated heat-stressed plants, but the transcription was lowered more sharply in HT-treated plants.

**Figure 5:**
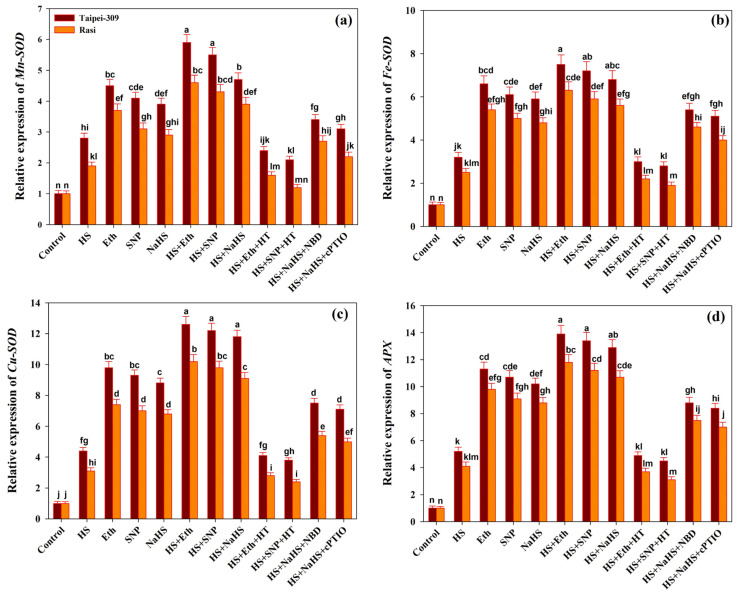
Relative expression of (**a**) *Mn-SOD*, (**b**) *Fe-SOD*, (**c**) *Cu-SOD*, and (**d**) *APX* of rice (*Oryza sativa* L.) cultivars Taipei-309 and Rasi under control and high temperature stress (HS) supplied with 200 µL L^−1^ ethephon (Eth), 100 µM sodium nitroprusside (SNP), 200 µM sodium hydrosulfide (NaHS) or 100 µM hypotaurine (HT), 100 µM 2-4-carboxyphenyl-4,4,5,5-tetramethylimidazoline-1-oxyl-3-oxide (cPTIO), or 100 µM norbornadiene (NBD) scavengers of hydrogen sulfide (H_2_S), nitric oxide (NO), and ethylene action inhibitors, respectively. Data are presented as treatments mean ± SE (*n* = 4). The values followed by the same letters did not differ significantly by LSD test at *p* < 0.05.

The relative expression analysis based on a qRT-PCR method for three SOD isoforms containing *Mn-SOD*, *Cu-SOD*, and *Fe-SOD* and *APX* was carried out on two rice cultivars (Figure 5). The results revealed that the expression of SOD isoforms and *APX* was upregulated in HS-treated rice cultivars compared to control plants. The individual application of Eth, SNP, or NaHS further enhanced the expression of SOD isoforms and APX in rice cultivars exposed to HS compared to heat-treated plants alone.

Meanwhile, in HT treatment with Eth or SNP under HS, no significant difference was observed in the expression levels of SOD isoforms and *APX* compared to control plants. Unlike the above, Eth, SNP, or NaHS treatments significantly increased the transcription level of these genes in heat-exposed plants. In contrast, HT application appears to affect antioxidant defense-related gene expression.

### 3.10. Determination of Interaction among Morpho-Physiological, Biochemical and Molecular Parameters through PCA

**Figure 6:**
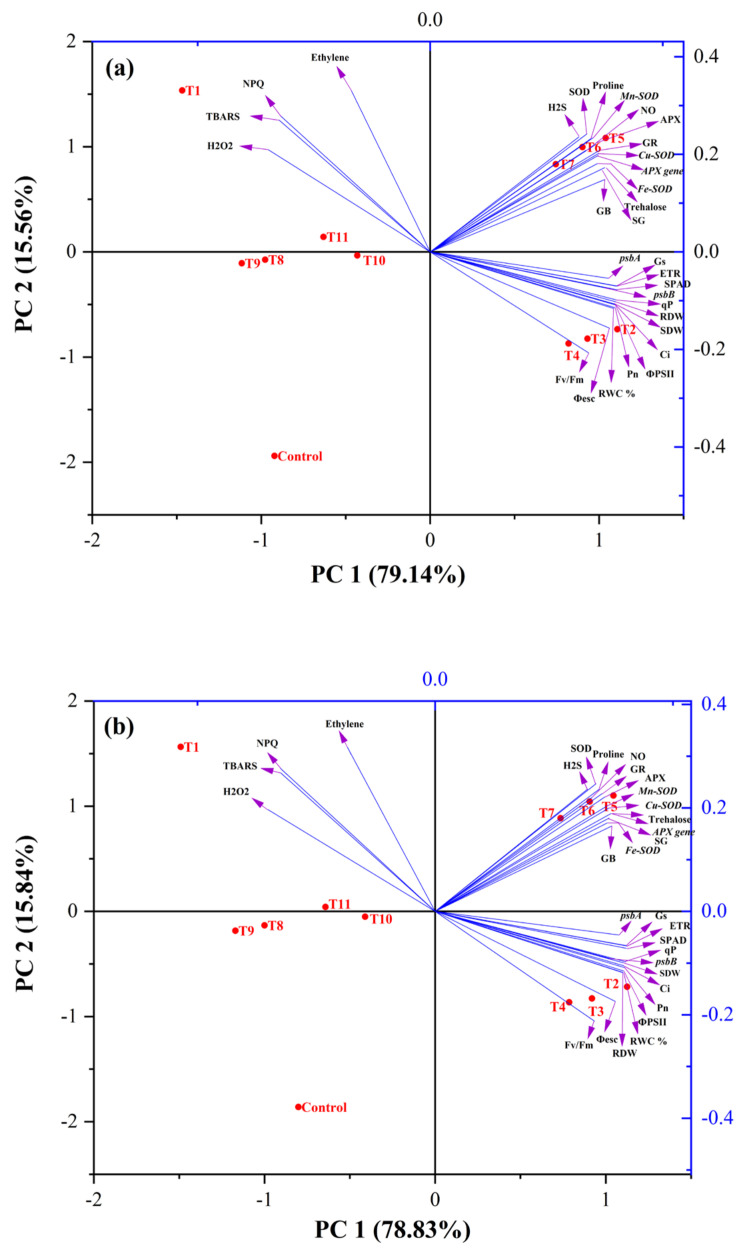
Biplots of principal component analysis (PCA) represents the relationship among different treatments and variables of two rice cultivars, Taipei-309 (**a**) and Rasi (**b**) grown under different conditions such as control, high temperature stress; HS (T1), Eth; Eth (T2), sodium nitroprusside; SNP (T3), sodium hydrosulfide; NaHS (T4), HS + Eth (T5), HS + SNP (T6), HS + NaHS (T7), HS + Eth + hypotaurine; HT (T8), HS + SNP + HT (T9), HS + NaHS + norbornadiene; NBD (T10), and HS + NaHS + 2-4-carboxyphenyl-4,4,5,5 -tetramethylimidazoline-1-oxyl-3-oxide; cPTIO (T11). The variables included H_2_O_2_ (hydrogen peroxide), TBARS (thiobarbituric acid reactive substances), non-photochemical quenching (NPQ), ethylene evolution, SOD (superoxide dismutase), APX (ascorbate peroxidase), GR (glutathione reductase) activity, gene expression of (*Mn-SOD*, *Cu-SOD*, *Fe-SOD*, and *APX*), contents of H_2_S (hydrogen sulfide), NO (nitric oxide), proline, trehalose, SG (soluble sugar) and GB (glycine betaine), gene expression of *psbA* and *psbB*, *Pn* (net photosynthesis), *Gs* (stomatal conductance), *Ci* (intercellular CO_2_ concentration), SPAD value, Fv/Fm (maximum efficiency of PSII), ΦPSII (actual efficiency of PSII), Φesc (intrinsic efficiency of PSII), qP (photochemical quenching), NPQ (non-photochemical quenching), ETR (electron transport rate), RDW (root dry weight), SDW (shoot dry weight), and RWC% (relative water content).

The PCA was carried out to determine the degree of data variation and the relationship between the different treatments and parameters in both rice cultivars (Figure 6). The two components (PC1 and PC2) described 94.70% of data variability in Taipei-309 under the influence of different treatments (Figure 6). PC1, the first component, contributed 79.14% of the total variation, and the second component, PC2, accounted for 15.56% of the total variation. On the other hand, in the Rasi cultivar, the PCA showed 94.67% (PC1 = 78.83% and PC2 = 15.84%) data variance under the influence of different treatments (Figure 6. Shoot and root dry weight, SPAD value, parameters of chlorophyll fluorescence (ETR, qP, ΦPSII, Φesc, and Fv/Fm), net photosynthesis, intercellular CO_2_ concentration, stomatal conductance, photosynthesis-related genes (*psbA* and *psbB*), and RWC were grouped together and exhibited a positive correlation. Antioxidant enzymes (SOD, APX, and GR) and genes (*Mn-SOD*, *Cu-SOD*, *Fe-SOD*, and *APX*), contents of proline, GB, soluble sugars and trehalose, NO, and H_2_S content were grouped together and were also found to bepositively correlated with plant dry mass, chlorophyll fluorescence parameters, and photosynthesis parameters. However, H_2_O_2_ and TBARS, NPQ, and ethylene contents were grouped together and showed a negative correlation with other groups of parameters.

### 3.11. Pearson Correlation

**Figure 7:**
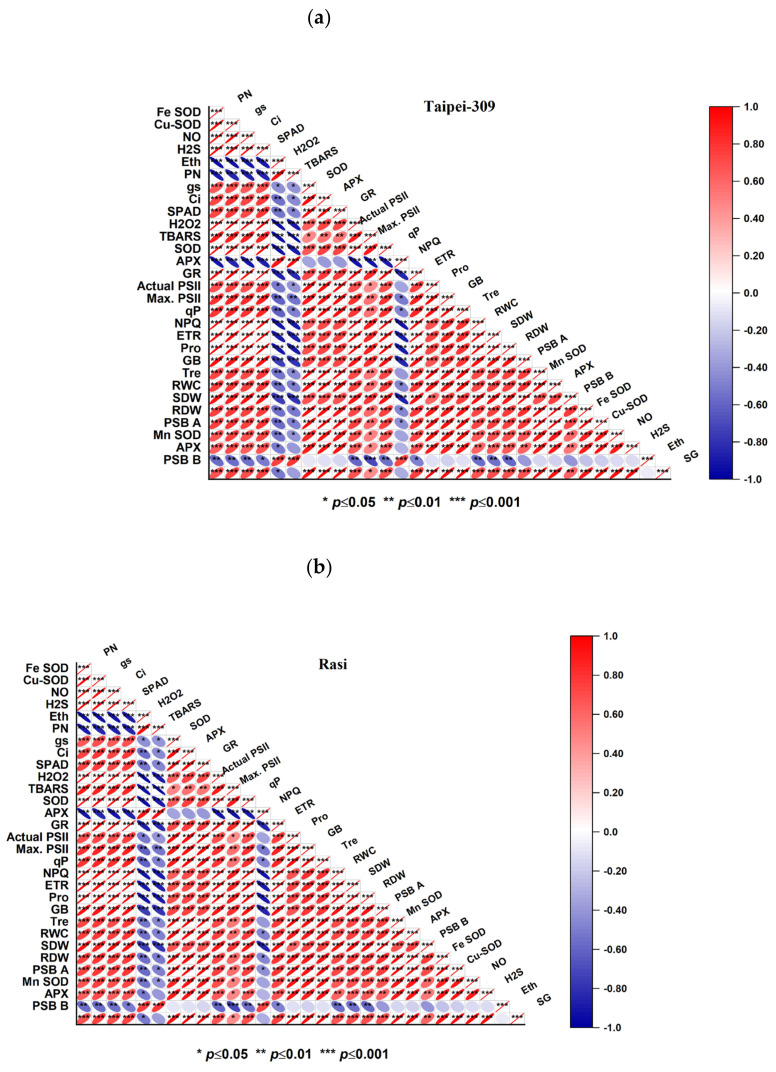
Pearson correlation represents the relationship among different variables of two rice cultivars, Taipei-309 (**a**) and Rasi (**b**) grown under different conditions such as control, high temperature stress; HS (T1), Eth; Eth (T2), sodium nitroprusside; SNP (T3), sodium hydrosulfide; NaHS (T4), HS + Eth (T5), HS + SNP (T6), HS + NaHS (T7), HS + Eth + hypotaurine; HT (T8), HS + SNP + HT (T9), HS + NaHS + norbornadiene; NBD (T10) and HS + NaHS + 2-4-carboxyphenyl-4,4,5,5 -tetramethylimidazoline-1-oxyl-3-oxide; cPTIO (T11). The variables included H_2_O_2_ (hydrogen peroxide), TBARS (thiobarbituric acid reactive substances), non-photochemical quenching (NPQ), ethylene evolution, SOD (superoxide dismutase), APX (ascorbate peroxidase), GR (glutathione reductase) activity, gene expression of (*Mn-SOD*, *Cu-SOD*, *Fe-SOD*, and *APX*), contents of H_2_S (hydrogen sulfide), NO (nitric oxide), proline, Tre (trehalose), SG (soluble sugar) and GB (glycine betaine), gene expression of *psbA* and *psbB*, *Pn* (net photosynthesis), *Gs* (stomatal conductance), *Ci* (intercellular CO_2_ concentration), SPAD value, maximum efficiency of PSII, actual efficiency of PSII, qP (photochemical quenching), NPQ (non-photochemical quenching), ETR (electron transport rate), RDW (root dry weight), SDW (shoot dry weight), and RWC (relative water content).

The Pearson correlation heatmap of Taipei-309 and Rasi showed a strong linear correlation of NO, ethylene, and H_2_S with growth, leaf water status, osmolytes, antioxidants, and the photosynthesis of plants (Figure 7). The plant growth and photosynthesis variables showed a negative correlation with the high temperature-induced oxidative stress biomarkers like H_2_O_2_ and TBARS content. The content of NO, H_2_S, GB, Tre, SG, and Pro showed a significant (*p* ≤ 0.05, *p* ≤ 0.01, and *p* ≤ 0.001) positive correlation with plant growth (SDW and RDW), photosynthetic parameters (SPAD, PN, gs, Ci, Actual PSII, Maximum PSII, qP, and ETR), the expression of photosynthesis-related genes (PSB A and PSB B), and the level of antioxidant enzymes. On the other hand, endogenous Eth showed a strong correlation with oxidative stress, suggesting its potential role and generation during stress conditions. The photosynthesis-related genes showed a strong dependency upon the relative expression of *Mn-SOD*, *Cu-SOD*, and *Fe-SOD* and *APX*. Therefore, these connections portray a nearby association between NO, Eth, and H_2_S and plant response to thermo-tolerance in Taipei-309 and Rasi cultivars.

## 4. Discussion

High temperature stress, one of the most common types of abiotic stresses that plants face in nature, has an independent mode of action on the physiology and metabolism of plant cells. Previous studies have documented the effects of the application of several signaling molecules and growth regulators on various plant species; however, there aren’t many reports on the comparative actions of ethylene, NO, and H_2_S on rice cultivars under HS. In the current study, we evaluated the efficacy of ethylene, NO, and H_2_S in modulating photosynthesis, growth, osmolytes, antioxidant metabolism, and the potential to ameliorate oxidative stress-induced impairments in rice cultivars subjected to HS. Among the various treatments used, 200 µL L^−1^ ethylene treatments were the most effective, followed by 100 µM SNP and 200 µM NaHS. Meanwhile, we also explored the influence of H_2_S in ethylene or NO-mediated tolerance of HS in rice cultivars.

The findings of the present study revealed that HS negatively impacts growth parameters and photosynthesis, which could be linked to an elevated level of oxidative stress indicators in rice plants, as evident from PCA and Pearson correlation. High temperature stress reduced the SPAD value, stomatal conductance, intercellular CO_2_ concentration, and net photosynthetic rate. Heat stress has been observed to cause changes in plant growth, pigment concentrations, and photosynthesis in various plants [3,4,11,49]. Furthermore, previous research has found that severe heat stress in plants can result in cellular injury, cell death, and a reduction in the total dry weight of plants [57,58]. To examine whether or not Eth, SNP, or NaHS can mitigate the detrimental effect of HS on plant growth attributes and photosynthetic activity, they were sprayed onto the foliage of rice plants. The result showed that Eth, SNP, or NaHS relatively relieved reduced plant growth and photosynthesis. Overall, the maximum improvement in plant growth and photosynthesis was recorded from the plants treated with Eth followed by SNP and NaHS treatments. In comparison to SNP and NaHS, Eth might be an active growth regulator involved in the heat tolerance of rice cultivars. To understand more about whether H_2_S plays a role in ethylene or NO-induced heat tolerance of rice cultivars, HT, an H_2_S scavenger, was given to the plants treated with Eth or SNP under HS stress. The results of this study showed that when HT was applied along with Eth or SNP, these treatments were ineffective in enhancing plant growth and photosynthesis when exposed to HS stress. HT reversed the availability of H_2_S, and Eth or NO were unable to efficaciously sustain heat tolerance in rice cultivars. Thus, the findings imply that ethylene or NO causes H_2_S to be produced in heat-stressed plants and that H_2_S increases heat tolerance in rice cultivars. Eth, NO, and H_2_S are important gaseous signaling molecules that regulate each other’s behavior, and H_2_S might work as a downstream signaling agent of NO and Eth on photosynthetic and growth under heat stress. In the present study, the supplementation of Eth, NO, and H_2_S effectively alleviated heat stress, which was reversed by the supplementation of HT, a H_2_S scavenger, suggesting that H_2_S works as a downstream signaling agent in NO and Eth-mediated heat stress tolerance. However, in the signaling cascade, H_2_S may act either upstream or downstream of NO, and there are complex relationships between NO, Eth, and H_2_S that are engaged in a variety of physiological processes and pathways.

Exogenously-applied H_2_S has been shown to promote growth and reduce lead (Pb) accumulation in *Zea mays* plants under Pb stress [59], improve photosynthesis, protect chloroplast structure, and promote growth in *Oryza sativa* under Ni stress [60], and improve the content of photosynthetic pigments and seedling biomass in *Cucurbita pepo* under nickel (Ni) stress [61]. H_2_S and NO are known to increase plant resistance to a variety of stresses, and they might serve as secondary signals to activate signal pathways downstream. [62,63]. The H_2_S has previously been shown to act as a downstream signal in the NO-induced enhanced adaptability of heat in maize plants [64]. The interaction of ethylene and H_2_S in heat stress tolerance has been shown [65].

Photosystem II is a pigment-protein complex with many components that are important for water splitting, oxygen evolution, and plastoquinone reduction. The photosystem PS II is more sensitive to environmental stress than PS I in chloroplasts [66,67]. Chlorophyll fluorescence parameters have been proven to be an effective measure of stress intensity [68]. The result of the present study demonstrated that chlorophyll fluorescence parameters were reduced in heat-exposed rice plants, which contributed to the decrease in net photosynthesis. Under HS stress, there was a decrease in ΦPS II, Fv/Fm, Φesc, ETR, and qP, as well as an increase in NPQ. This indicates that heat stress-induced ROS production causes a decrease in PS II reaction center activity and renders the reaction center unable to use light energy efficiently. Havaux [69] observed an irreversible decline in the photochemical efficiency after 90 min of exposure of *Solanum tuberosum* plants to 39.5 °C, whereas Camejo et al. [70] showed that heat-sensitive tomato (*Lycopersicon esculentum* Mill. cv. Campbell-28) plants exhibited a decline in Rubisco activity and PS II performance after exposure of plants to 45 °C for 2 h. Heat stress not only enhances thylakoid membrane fluidity but also causes protein complexes and photosystems to reorganize and even dissociate [71,72]. Extreme heat stress causes structural changes in protein complexes, photosystem degradation, and a loss of oxygen-evolving activity, all of which impair the photosystem’s ability to transfer electrons [73]. The application of Eth, SNP, or NaHS reversed photo-inhibition and the impairment of photosynthetic characteristics caused by HS. Furthermore, the application of HT along with Eth or SNP enhanced the detrimental effects of HS and reversed the mitigation effects of Eth or SNP on chlorophyll fluorescence attributes, implying that H_2_S plays an important role in regulating the impact of HS on photosynthetic attributes. As a result, H_2_S is implicated in the augmentation of PS II reaction center activity via ethylene or NO in rice cultivars subjected to heat stress, and it participated with ethylene or NO to improve light energy utilization efficiency. It has previously been demonstrated that ethylene application contributes to waterlogging stress reduction by strengthening photosynthetic pigment or improving electron transport [74]. According to Shi et al. [75], the Fv/Fm and ETR were higher in the presence of SNP, indicating that NO partially alleviated photodamage in UV-B-stressed bean leaves. In a previous study on the mung bean cultivars, it was reported that ethylene and H_2_S can protect photosynthesis against hexavalent chromium stress [37].

In this study, we assessed the RWC of rice cultivars under various treatments. Under HS stress, RWC declined significantly in both rice cultivars, with Rasi experiencing a greater decrease. Heat stress may reduce the water status of the leaves by reducing the hydraulic conductance, resulting in a decrease in water absorption, or by lowering stomatal conductance [76]. In this study, the heat-sensitive cultivar Rasi was shown to lose more water than Taipei-309, the heat-tolerant cultivar. Individual applications of Eth, SNP, or NaHS significantly altered the RWC of rice cultivars in which Eth showed better results than SNP and NaHS treatments. Furthermore, the applications of Eth, SNP, or NaHS enhanced the RWC of leaves under HS, thereby reducing heat-related plant damage. Intriguingly, the treatment of HT with Eth or SNP reversed this effect, suggesting that H_2_S was involved in ethylene or NO-induced changes in RWC in rice cultivars under HS stress. NO treatment, according to Khan et al. [77], benefitted mustard plants in retaining more water when subjected to salt stress. Similarly, Li et al. [78] observed that H_2_S maintained leaf RWC in cadmium (Cd)-stressed seedlings of *Brassica rapa*. Higher leaf RWC may have enhanced stomatal conductivity and, as a result, photosynthetic activity and biomass production [79]. Tomato plants treated with NaHS or Eth showed no decrease in RWC in response to low osmotic stress but did show a slight decrease in response to severe osmotic stress [38].

Many organisms counteract the environmental challenges by accumulating low molecular weight water-soluble substances known as osmolytes. Under heat stress, the accumulation of osmolytes aids in osmotic adjustment increases the concentration of cell protoplasm to maintain proper membrane function and quenches ROS in plants. [80,81]. The reason for higher proline levels is related to the synthesis and accumulation of free amino acids under stressed conditions [82]. Furthermore, GB accumulated in transgenic tobacco plants improved PS II thermo-tolerance from heat stress [83]. Osmolytes serve as stress markers and hence play an important role in stress reduction. *Zea mays* L. plants subjected to copper (Cu) and Pb exhibited higher levels of proline, which protected them from an oxidative burst and helped to maintain cell structures [84]. Heat tolerance, a crucial physiological trait for heat resistance, necessitates the accumulation of sugars in plants and the availability of carbohydrates [85]. A non-reducing disaccharide known as trehalose accumulated in Arabidopsis plants exposed to heat stress and served as a ROS scavenger in heat-exposed wheat plants [86,87]. Trehalose is essential for maintaining growth under adverse conditions because it controls how efficiently most plants use water and stomatal movement [88]. According to Li et al. [89] trehalose serves as an osmoprotectant during water deficit, which aids in stabilizing dehydrated enzymes, proteins, and membrane lipids and guards against damage to biological structures.

The results of the present study showed that heat stress treatment increased the levels of osmolytes such as proline, GB, trehalose, and soluble sugars; however, these increased levels of osmolytes were unable to counteract heat stress and settled the stressed rice cultivars with decreased water status. However, the application of Eth, SNP, and NaHS to stressed rice cultivars augmented proline, GB, and trehalose levels, which reduced heat stress and enabled plant cells with increased osmotic pressure to take in more water as evidenced by enhanced RWC. On the other hand, the application of HT with Eth or SNP under heat stress lowered the amount of these osmolytes, confirming the role of H_2_S in the ethylene- or NO-mediated osmotic adjustment of plants. Previously, it was shown that NO-induced H_2_S generated an increase in proline and GB, which protected wheat plants from osmotic stress-induced oxidative stress [90]. The observed augmentation of proline, GB, total soluble sugars, and total soluble proteins in response to NO application potentially improved salt tolerance through osmotic regulation [77]. Under heat stress and after applying ABA and NO, trehalose accumulation increased even more [91]. In *P. eryngiivar. tuoliensis* under heat stress, trehalose accumulation increased with NO [92]. Heat-stressed rice cultivar leaves accumulated higher levels of H_2_O_2_ and TBARS contents. Increased levels of ROS may be attributed to the altered photosynthetic process in rice cultivars under heat stress. The enhanced ROS levels were accompanied by increased lipid peroxidation. However, Eth, SNP, or NaHS application resulted in a reduction in the levels of H_2_O_2_ and TBARS in both heat-stressed cultivars, more effectively in Taipei-309. Therefore, the application of Eth, SNP, or NaHS could be a useful strategy to prevent plants from oxidative damage brought on by HS. These results show that Eth treatment alleviated heat-induced oxidative damage more effectively than SNP and NaHS in rice cultivars by lowering H_2_O_2_ and TBARS contents. An excess in ROS could cause severe damage to lipids and proteins, which is a major cause of plant growth reduction [93]. However, using HT with Eth or SNP dramatically reversed this effect and resulted in considerable cell membrane damage exhibited as a significant increase in TBARS and H_2_O_2_ levels. The elimination of H_2_S via scavenging resulted in the production of ROS again. It reveals that ethylene or NO-induced H_2_S were involved in reducing stress intensity in plants by scavenging ROS and decreasing lipid peroxidation, hence minimizing oxidative damage. Moreover, investigations have shown that individual applications of ethylene or H_2_S have the capacity to reduce the levels of ROS in plants under abiotic stress [94,95]. The treatment of NO has an important role in enhancing endogenous H_2_S production, which helps plants resist abiotic stress-induced oxidative stress by reducing ion leakage, H_2_O_2_, O_2_^−^, and TBARS levels [96,97,98]. NO has been found to reduce lipid peroxidation and ROS production in plants grown in Cd and Cu-enriched environments [23,99].

The result showed that HS resulted in oxidative stress as observed by the excessive production of ROS. Overproduction of ROS occurs in stressed cells when the cellular antioxidant defense mechanism is slower than the ROS synthesis that causes oxidative stress. In the present study, under HS, the activities of antioxidant enzymes, APX, GR, and SOD were enhanced; simultaneously, the H_2_O_2_ and TBARS content also increased in both the cultivars. As a result, increased levels of antioxidant enzymes in heat-stressed plants were insufficient to detoxify ROS, resulting in an excess of H_2_O_2_ and TBARS accumulation. Meanwhile, the application of Eth, SNP, or NaHS to heat-stressed plants increased antioxidant enzyme activity to the point where it was capable of detoxifying ROS by significantly lowering the levels of H_2_O_2_ and TBARS. Furthermore, in heat-challenged plants, the treatment of HT with Eth or SNP caused an alteration in the antioxidant enzyme activities induced by Eth or SNP. According to the present study, the exogenous application of SNP or Eth induced H_2_S production and improved heat stress tolerance, which could be altered by treatment with an HT, suggesting that NO or ethylene-activated H_2_S might be required for heat stress response in rice plants. Under stress, the levels of ROS are tightly regulated by enzymatic and non-enzymatic antioxidants, determining the stressed plant’s fate. The enzyme SOD is well-known for dismutating superoxide (O_2_^−^ radicals to hydrogen peroxide (H_2_O_2_), whereas APX and GR transform H_2_O_2_ to water and oxygen. An increase in H_2_O_2_ levels due to an inhibition in APX activity could damage lipids and proteins [98]. Exogenous NO may stimulate the synthesis of endogenous NO, that can function as a signaling molecule or ROS scavenger under intense stress circumstances by controlling and improving the activities of antioxidant enzymes [100,101]. Through explorations into the effects of exogenous H_2_S in wheat during flooding-induced hypoxic stress, it has been reported that NaHS application positively enhanced the activity of certain enzymes, including APX and GR [102]. In arsenate-only treated seedlings, the addition of NaHS raised NO levels, implying that both (H_2_S and NO) cause the upregulation of the ascorbate-glutathione (AsA and GSH) cycle to counterbalance ROS-mediated damage, resulting in enhanced pea seedling growth according to Singh et al. [98]. Additionally, NO plays a crucial role in encouraging endogenous H_2_S production, which increases the activity of antioxidant enzymes and aids wheat plants in tolerating oxidative stress brought on by osmotic stress [92]. Furthermore, NO and H_2_S promote protein post-translational modifications via S-nitrosylation and tyrosine nitration. The altered protein function and activity caused by such Pb modifications may have given plants greater tolerance to abiotic stress [103,104]. Consistent with the accumulation of antioxidant enzymes in rice cultivars subjected to heat stress, either alone or in combination with Eth, SNP, or NaHS, the expression of SOD isoforms (*Mn-SOD*, *Fe-SOD*, *Cu-SOD*) and *APX* genes were also upregulated in treated rice cultivars. This suggested that up-regulation of *SOD* isoforms and *APX* genes could improve the activities of the SOD and APX enzymes, hence protecting cells from oxidative damage caused by HS stress. The activity of SOD isoenzymes is increased by ethylene in Arabidopsis plants under Cd stress, which affects root morphology [105]. In EIN2-1 mutant plants, higher transcription levels of Cu/Zn SOD2 and CAT3 resulted in higher SOD and CAT enzyme activity when compared to control plants [106,107]. In contrast, other investigations have revealed that inducing ethylene under abiotic stress could be harmful to plants, decreasing the activity of antioxidative enzymes and increasing the accumulation of ROS [108,109]. The hormone, NO’s antioxidant property may be due to its direct interaction with ROS, which is then neutralized by several cellular processes, or NO could boost the antioxidant potential of cells by enhancing antioxidant enzyme activities [110]. At the post-translational level, NO modulates APX through S-nitrosation of cysteine residues, which enhances its activity, and metal nitrosation and tyrosine nitrosation, which both decrease its activity [111,112]. In a prior study, NaHS root pretreatment boosted the gene expression of antioxidant enzymes (cAPX, CAT, Mn-SOD, and GR), heat shock proteins (HSP70, HSP80, and HSP90), and aquaporins (PIP) [113]. Furthermore, we found that HT treatment with Eth or SNP under HS stress reversed Eth- or SNP-induced upregulation of *SOD* isoforms and *APX* genes. In *Solanum lycopersicum*, ethylene and H_2_S fumigation sustained higher levels of *SlAPX1*, *SlAPX2*, and *SlCAT3* expression [39].

A previous study indicated that HS stress may enhance NO synthesis in tobacco [114] and higher plants [115]. Similarly, in this study, HS stress increased NO levels in rice cultivar leaves. Furthermore, plants under HS stress had higher levels of H_2_S in their leaves. Similar to these results, increased H_2_S generation was seen in wheat [36] and maize [116] exposed to heat stress, as well as bermudagrass exposed to cold, salt, and osmotic stresses [87]. In our experimental conditions, a rise in both NO and H_2_S levels under HS stress was detected, which is in good agreement with these reports. In the present study, donors and inhibitors of H_2_S and NO were applied to rice cultivars subjected to heat stress in order to better understand the interaction between H_2_S and NO. In this study, NaHS, SNP, and Eth treatment raised NO content in rice cultivar leaves in both heat-stressed and no-stress conditions; such an increase has previously been documented in barley and wheat plants [117,118]. Under HS stress, the decrease in NO content was greatest when HT was combined with Eth or SNP, which affected stress alleviation. Research indicates an interaction between NO and H_2_S and it was recently reviewed [119]. Similarly, SNP, Eth, or NaHS treatment improved the H_2_S content in leaves of rice cultivars in control and stressed plants, but significantly with NaHS treatment. Furthermore, the suppression of ethylene and NO in the presence of NaHS under HS stress using their inhibitors NBD and cPTIO, respectively, had no significant influence on H_2_S levels.

Ethylene is produced in response to a variety of environmental stresses, implying that it acts as a connection between environmental change and developmental adaptability [120]. Ethylene increases photosynthesis and dry matter accumulation in plants under optimal and stressful environments. However, ethylene homeostasis is important for plant response and stress tolerance since excess ethylene formation under stress condition negatively impacts plant physiological and metabolic functions and plant growth. The involvement of ethylene in heat stress tolerance has been investigated earlier [17,49]. Previous studies have shown that heat stress, particularly in the 30–38 °C range, causes an increase in ethylene production in plants such as *Phaseolus vulgaris* [121] and *Triticum aestivum* [122]. Salt tolerance depends on ethylene production, and ethylene signaling is crucial for plants to self-correct quickly in response to salinity stress and to adapt better to the stress condition [123]. Under heat stress conditions, plants release stress ethylene by the same process that produces ethylene during normal development. In the present study, the ethylene level in heat-stressed plants was higher than control plants because of the burst of ethylene that occurred under stress. The application of ethephon following heat treatment resulted in ethylene release that showed protective functions at this stage and induced mechanisms of the activation of the antioxidant system to scavenge ROS and relieve plants from the stress. As the plants were relieved from the stress, the burst of stress ethylene was minimized resulting in a lower level of ethylene compared to heat-stressed plants. It has been shown that when plants are exposed to conditions that threaten their ability to survive, the same mechanism that produces ethylene for normal development instead functions to produce what is known as stress ethylene [124]. The paradoxical effects of stress ethylene on plants were shown emphasizing the fact that in stressed plant tissues, there is an initial small peak of ethylene close in time to the onset of stress and the second much larger peak some time later. The first small peak shows the protective response of plant. The second peak is so large that processes that are inhibitory to plant survival are initiated [124]. Thus, the modulation of ethylene production could reduce the stress-related injuries. According to studies, the signaling molecule NO modulates endogenous ethylene levels at different levels by altering a variety of pathways, leading to post-climacteric biochemical changes related to fruit quality [125]. In the current study, the application of SNP or NaHS resulted in lower ethylene levels more notably when there was no stress, compared to HS plants. It was recently proposed that H_2_S counteracts the effect of ethylene action in banana fruit ripening and senescence [126]. As a result, H_2_S might be able to resist ethylene function. Furthermore, H_2_S, which is similar to NO, inhibits 1-amino-cyclopropane carboxylic acid oxidase (ACO) activity in tomato leaves [127]. In this study, we used HT to investigate the mechanism of H_2_S and its effect on ethylene levels. Under HS stress, the application of HT with Eth or SNP increased the level of ethylene.

Furthermore, HS conditions have been shown to affect the light-harvesting complex, water-oxidizing complex, and PS II reaction center [128]. Chloroplast gene expression and responses to environmental stress may be related [31]. The chloroplast genes *psbA* and *psbB* encode the D1 protein of PS II and the PS II chlorophyll-binding protein (CP47), respectively [129,130]. In the present study, the qRT-PCR analysis revealed that heat stress downregulated the expression of the *psbA* and *psbB* genes, which were linked to PS II inactivation. Reduced photosynthetic pigments and organic solutes, such as soluble sugars, sucrose, and proline, were associated with the deleterious consequences of HS stress [131]. Salt stress was reported to cause the degradation of D1 protein (encoded by the *psbA* gene) in *Avena sativa* plants, as well as the downregulation of *psbA*, *psbB*, *psbC*, and *psbD* [132]. Meanwhile, Eth, SNP, or NaHS treatments upregulated the *psbA* and *psbB* gene expression of heat-stressed rice cultivar leaves, which may be responsible for PS II stability under heat stress. Notably, the expression of the investigated genes was significantly higher in Eth-treated leaves compared to SNP and NaHS leaves. Photosystem II tolerance to high light would be improved by enhanced *psbA* transcription and translation [31]. On the contrary, the application of HT along with Eth or SNP under HS resulted in the downregulation of *psbA* and *psbB* gene expression. It was investigated that H_2_S was involved in the ethylene- or NO-mediated protection of photosynthetic machinery during HS stress, hence enhancing photosynthetic efficiency and mitigating the negative consequences of HS stress.

**Figure 8:**
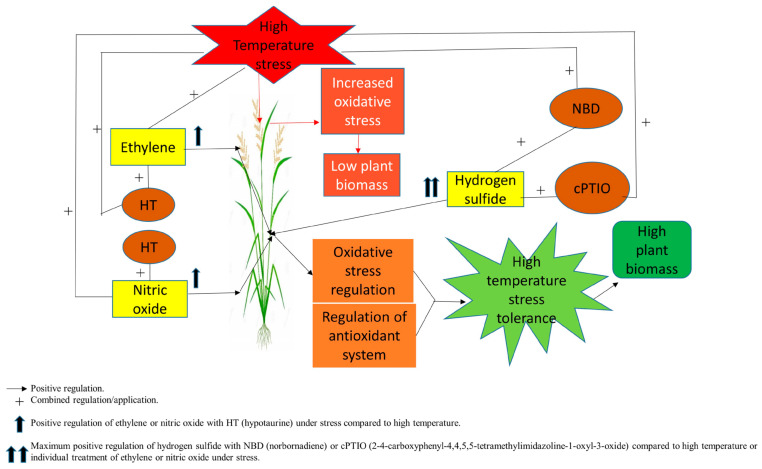
Proposed model for the role of ethylene, NO and H_2_S in the alleviation of high temperature stress in rice (*Oryza sativa* L.) NBD; norbornadiene, cPTIO; 2-4-carboxyphenyl-4,4,5,5-tetramethylimidazoline-1-oxyl-3-oxide, HT; hypotaurine.

In the present study, the data were also examined using PCA in order to identify and classify the enormous data set in terms of growth, physio-biochemical and molecular characteristics into a manageable set of dynamically interrelated variables [133,134,135]. The PCA explained 94.70% and 94.67% of the data variability in Taipei-309 and Rasi, respectively, which accords with Sneath and Sokal [136], who considered that data must account for at least 70% of the total variability. The lines originating from the central point of the biplot represent correlations between various variables, with the closeness of the lines indicating the strength of the correlation with a specific treatment. Treatments such as (HS + Eth + HT), (HS + SNP + HT), and control were distributed in left quadrant, i.e., in the negative direction. However, treatments such as Eth, SNP, and NaHS with no stress and HS stress were present away from the origin in a positive direction. Growth and physiological attributes clustered opposite to oxidative stress attributes and NPQ. Biplot categorized the traits into three groups. The first group provided three treatments, Eth, SNP, and NaHS, under control conditions. These treatments were correlated to the variables including the dry weight of root and shoot, chlorophyll fluorescence, photosystem II genes, photosynthetic traits, and leaf RWC. This suggests that under control conditions, these treatments significantly increased these traits. Afterward, Eth, SNP, and NaHS under HS were grouped together. These treatments have a significant association with antioxidant enzyme activity (SOD, APX and GR), antioxidant genes (*Mn-SOD*, *Cu-SOD*, *Fe-SOD*, and *APX*), and osmolytes (proline, GB, soluble sugar, and trehalose), NO, and H_2_S content. In addition, HS treatment shows a significant association with oxidative stress markers (H_2_O_2_ and TBARS), NPQ, and ethylene levels. However, treatments with inhibitors and scavengers such as HT (HS + Eth + HT) and (HS + SNP + HT), NBD (HS + NaHS + NBD) and cPTIO (HS + Eth + cPTIO) seemed to have no significant association with any parameter. This analysis confirmed our results that HS stress reduced the majority of morpho-physiological and biochemical traits while increasing H_2_O_2_ and TBARS content and NPQ in the two cultivars tested. On the other hand, Eth, SNP, and NaHS increased most of the traits under control and heat conditions. In contrast, the application of HT did not demonstrate any significant association with any parameters A proposed model to show the significance of ethylene, NO and H_2_S in high temperature stress tolerance is presented in Figure 8.

To further confirm the role of ethylene and NO in H_2_S-mediated thermotolerance, we run our dataset for Pearson correlation. The correlation between all paired attributes of antioxidant enzymes, their relative expression and osmolytes were positively significant with growth and photosynthesis parameters and negatively correlated with oxidative stress markers.

## 5. Conclusions

In summary, signaling molecules such as ethylene, H_2_S, and NO have been found as promising for enhancing rice plants’ thermo-tolerance, as well as growth and photosynthesis, particularly more in Taipei than Rasi. However, in both rice cultivars, Eth was more effective than SNP and NaHS in alleviating HS. The activation of antioxidant enzymes, the detoxification of ROS, and the higher accumulation of osmolytes caused by the use of signaling molecules equipped the plants in combating the negative effects of heat stress. In addition, the scavenging of H_2_S by HT subsequently damaged the rice plants in the presence of Eth or SNP, confirming that the beneficial action of Eth and SNP is, at least to some extent, reliant on H_2_S.

## Figures and Tables

**Table 1:** Net photosynthetic rate (µmol CO_2_ m^−2^ s^−1^), stomatal conductance (mmol m^−2^ s^−1^), intercellular CO_2_ concentration (µmol mol^−1^), and chlorophyll content (SPAD value) of rice (*Oryza sativa* L.) cultivars Taipei-309 and Rasi after foliar treatment of plants with 200 µL L^−1^ ethephon (Eth), 100 µM sodium nitroprusside (SNP) or 200 µM sodium hydrosulfide (NaHS) grown with or without high temperature stress (HS; 40 °C) or 100 µM hypotaurine (HT), 100 µM 2-4-carboxyphenyl-4,4,5,5-tetramethylimidazoline-1-oxyl-3-oxide (cPTIO) or 100 µM norbornadiene (NBD) scavengers of hydrogen sulfide (H_2_S), nitric oxide (NO), and ethylene action inhibitors, respectively, with HS at 15 days after sowing. Data are presented as treatments mean ± SE (*n* = 4). The values followed by the same letters did not differ significantly by LSD test at *p* < 0.05.

	Taipei-309				Rasi			
Treatments	Net Photosynthetic Rate	Stomatal Conductance	Intercellular CO_2_ Concentration	SPAD	Net Photosynthetic Rate	Stomatal Conductance	Intercellular CO_2_ Concentration	SPAD
Control	15.1 ± 1.08 ^defg^	367.2 ± 15.0 ^de^	260.1 ± 13.8 ^de^	33.7 ± 1.30 ^g^	14.4 ± 1.04 ^fgh^	355.3 ± 14.0 ^ef^	251.2 ± 12.4 ^ef^	31.5 ± 1.25 ^gh^
HS	9.8 ± 0.66 ^ij^	268.3 ± 12.2 ^gh^	199.8 ± 11.6 ^gh^	24.1 ± 1.10 ^kl^	9.2 ± 0.55 ^j^	254.8 ± 11.0 ^h^	190.3 ± 10.2 ^h^	22.3 ± 1.0 ^l^
Eth	21.7 ± 1.45 ^a^	475.2 ± 16.5 ^a^	346.3 ± 14.9 ^a^	50.6 ± 1.76 ^a^	20.1 ± 1.28 ^abc^	451.1 ± 15.7 ^abc^	328.2 ± 14.5 ^abc^	46.2 ± 1.65 ^bcd^
SNP	21.3 ± 1.40 ^ab^	471.2 ± 16.4 ^ab^	340.3 ± 14.8 ^ab^	49.3 ± 1.69 ^ab^	19.7 ± 1.25 ^abc^	445.4 ± 15.6 ^abc^	320.4 ± 14.2 ^abc^	45.1 ± 1.51 ^bcde^
NaHS	20.9 ± 1.34 ^abc^	469.3 ± 16.0 ^ab^	339.6 ± 14.6 ^ab^	48.7 ± 1.60 ^abc^	19.1 ± 1.19 ^abc^	442.4 ± 15.1 ^abc^	319.7 ± 13.8 ^abc^	44.3 ± 1.40 ^cdef^
HS + Eth	19.5 ± 1.24 ^abc^	461.3 ± 15.9 ^abc^	315.6 ± 14.4 ^abc^	46.2 ± 1.55 ^bcd^	18.1 ± 1.13 ^bcd^	437.4 ± 14.9 ^bcd^	298.3 ± 13.7 ^bcd^	42.8 ± 1.37 ^def^
HS + SNP	19.2 ± 1.13 ^abc^	459.3 ± 15.9 ^abc^	312.9 ± 14.1 ^abc^	45.8 ± 1.45 ^bcd^	17.9 ± 1.10 ^bcde^	433.2 ± 14.8 ^bcd^	296.2 ± 13.6 ^bcd^	41.4 ± 1.35 ^ef^
HS + NaHS	18.8 ± 1.11 ^abc^	457.1 ± 15.4 ^abc^	311.9 ± 14.0 ^abc^	44.5 ± 1.40 ^cde^	17.4 ± 1.06 ^cdef^	431.6 ± 14.2 ^cd^	292.0 ± 12.4 ^cd^	40.2 ± 1.30 ^f^
HS + Eth + HT	13.1 ± 0.90 ^ghi^	311.6 ± 13.8 ^efgh^	230.8 ± 12.9 ^efgh^	29.4 ± 1.19 ^hij^	12.2 ± 0.85 ^ghij^	294.4 ± 12.9 ^efgh^	220.8 ± 11.3 ^efgh^	26.9 ± 1.13 ^ijk^
HS + SNP + HT	12.2 ± 0.83 ^ghij^	302.6 ± 13.7 ^efgh^	226.3 ± 12.5 ^efgh^	28.1 ± 1.15 ^hijk^	11.4 ± 0.77 ^hij^	289.1 ± 12.5 ^fgh^	214.3 ± 11.2 ^fgh^	25.4 ± 1.11 ^jkl^
HS + NaHS + NBD	14.6 ± 0.96 ^efgh^	361.7 ± 14.9 ^ef^	247.7 ± 13.7 ^ef^	31.9 ± 1.25 ^gh^	13.7 ± 0.91 ^gh^	341.2 ± 13.7 ^efgh^	233.8 ± 12.1 ^efgh^	29.1 ± 1.22 ^hij^
HT + NaHS + cPTIO	14.3 ± 0.92 ^fgh^	340.5 ± 14.7 ^efg^	242.7 ± 13.4 ^efg^	30.6 ± 1.20 ^ghi^	13.3 ± 0.88 ^gh^	323.1 ± 13.6 ^efgh^	227.8 ± 11.4 ^efgh^	28.2 ± 1.19 ^hijk^

**Table 2:** Actual efficiency of PSII, maximal efficiency of PSII, intrinsic efficiency of PSII, photochemical quenching, non-photochemical quenching, and electron transport rate of rice (*Oryza sativa* L.) cultivar Taipei-309 after foliar treatment of plants with 200 µL L^−1^ ethephon (Eth), 100 µM sodium nitroprusside (SNP), or 200 µM sodium hydrosulfide (NaHS) grown with or without high temperature stress (HS; 40 °C) or 100 µM hypotaurine (HT), 100 µM 2-4-carboxyphenyl-4,4,5,5-tetramethylimidazoline-1-oxyl-3-oxide (cPTIO) or 100 µM norbornadiene (NBD) scavengers of hydrogen sulfide (H_2_S), nitric oxide (NO), and ethylene action inhibitors, respectively, with HS at 15 days after sowing. Data are presented as treatments mean ± SE (*n* = 4). The values followed by the same letters did not differ significantly by LSD test at *p* < 0.05.

Treatments	Actual Efficiency of PSII	Maximum Efficiency of PSII	Intrinsic Efficiency of PSII	Photochemical Quenching	Non-Photochemical Quenching	Electron Transport Rate
Control	0.621 ± 0.036 ^bcdef^	0.808 ± 0.051 ^bcdef^	0.731 ± 0.045 ^abcde^	0.684 ± 0.045 ^abcdefghi^	0.573 ± 0.032 ^cdefgh^	164.6 ± 9.4 ^e^
HS	0.497 ± 0.021 ^gh^	0.622 ± 0.029 ^gh^	0.620 ± 0.025 ^de^	0.569 ± 0.029 ^i^	0.778 ± 0.052 ^ab^	132.8 ± 6.8 ^fg^
Eth	0.751 ± 0.060 ^a^	0.876 ± 0.071 ^a^	0.849 ± 0.068 ^a^	0.831 ± 0.067 ^a^	0.455 ± 0.021 ^h^	241.3 ± 11.2 ^a^
SNP	0.744 ± 0.059 ^a^	0.869 ± 0.069 ^a^	0.841 ± 0.066 ^a^	0.824 ± 0.063 ^ab^	0.463 ± 0.025 ^gh^	237.6 ± 10.2 ^ab^
NaHS	0.732 ± 0.052 ^ab^	0.861 ± 0.065 ^ab^	0.837 ± 0.061 ^ab^	0.816 ± 0.060 ^abc^	0.469 ± 0.029 ^gh^	231.8 ± 10.1 ^abc^
HS + Eth	0.711 ± 0.048 ^abc^	0.849 ± 0.062 ^abc^	0.796 ± 0.058 ^abc^	0.796 ± 0.058 ^abcde^	0.582 ± 0.034 ^cdefgh^	220.7 ± 9.7 ^abcd^
HS + SNP	0.707 ± 0.045 ^abc^	0.836 ± 0.059 ^abc^	0.789 ± 0.051 ^abc^	0.787 ± 0.055 ^abcdef^	0.591 ± 0.036 ^cdefg^	216.5 ± 9.6 ^abcd^
HS + NaHS	0.701 ± 0.040 ^abc^	0.830 ± 0.053 ^abc^	0.781 ± 0.048 ^abcd^	0.773 ± 0.051 ^abcdefg^	0.598 ± 0.038 ^cdefg^	212.4 ± 9.5 ^abcd^
HS + Eth + HT	0.563 ± 0.028 ^efgh^	0.777 ± 0.039 ^efgh^	0.695 ± 0.035 ^abcde^	0.642 ± 0.036 ^efghi^	0.651 ± 0.046 ^bc^	147.6 ± 8.4 ^efg^
HS + SNP + HT	0.547 ± 0.024 ^fgh^	0.769 ± 0.034 ^fgh^	0.686 ± 0.033 ^abcde^	0.631 ± 0.033 ^fghi^	0.660 ± 0.048 ^bc^	141.9 ± 7.7 ^efg^
HS + NaHS + NBD	0.598 ± 0.033 ^cdefg^	0.795 ± 0.047 ^cdefg^	0.727 ± 0.042 ^abcde^	0.671 ± 0.043 ^bcdefghi^	0.623 ± 0.040 ^cd^	159.3 ± 8.8 ^ef^
HS + NaHS + cPTIO	0.582 ± 0.030 ^defgh^	0.786 ± 0.042 ^defgh^	0.721 ± 0.039 ^abcde^	0.664 ± 0.040 ^cdefghi^	0.639 ± 0.043 ^c^	152.4 ± 8.6 ^ef^

**Table 3:** Actual efficiency of PSII, maximal efficiency of PSII, intrinsic efficiency of PSII, photochemical quenching, non-photochemical quenching, and electron transport rate of rice (*Oryza sativa* L.) cultivar Rasi after foliar treatment of plants with 200 µL L^−1^ ethephon (Eth), 100 µM sodium nitroprusside (SNP), or 200 µM sodium hydrosulfide (NaHS) grown with or without high temperature stress (HS; 40 °C) or 100 µM hypotaurine (HT), 100 µM 2-4-carboxyphenyl-4,4,5,5 -tetramethylimidazoline-1-oxyl-3-oxide (cPTIO) or 100 µM norbornadiene (NBD) scavengers of hydrogen sulfide (H_2_S), nitric oxide (NO), and ethylene action inhibitors, respectively, with HS at 15 days after sowing. Data are presented as treatments mean ± SE (*n* = 4). The values followed by the same letters did not differ significantly by LSD test at *p* < 0.05.

Treatments	Actual Efficiency of PSII	Maximum Efficiency of PSII	Intrinsic Efficiency of PSII	Photochemical Quenching	Non-Photochemical Quenching	Electron Transport Rate
Control	0.611 ± 0.025 ^bcdefg^	0.797 ± 0.042 ^bcdefg^	0.721 ± 0.038 ^abcde^	0.671 ± 0.037 ^bcdefghi^	0.582 ± 0.035 ^cdefgh^	153.7 ± 8.6 ^ef^
HS	0.472 ± 0.015 ^h^	0.601 ± 0.021 ^h^	0.598 ± 0.018 ^e^	0.543 ± 0.021 ^i^	0.799 ± 0.055 ^a^	121.4 ± 6.4 ^g^
Eth	0.725 ± 0.040 ^ab^	0.854 ± 0.067 ^ab^	0.826 ± 0.066 ^ab^	0.811 ± 0.056 ^abcd^	0.471 ± 0.027 ^fgh^	220.1 ± 10.7 ^abcd^
SNP	0.719 ± 0.036 ^ab^	0.847 ± 0.062 ^ab^	0.820 ± 0.063 ^ab^	0.801 ± 0.054 ^abcd^	0.483 ± 0.031 ^efgh^	215.8 ± 10.3 ^abcd^
NaHS	0.709 ± 0.034 ^abc^	0.839 ± 0.060 ^abc^	0.814 ± 0.059 ^abc^	0.798 ± 0.051 ^abcde^	0.492 ± 0.033 ^defgh^	210.6 ± 9.5 ^bcd^
HS + Eth	0.687 ± 0.032 ^abcd^	0.825 ± 0.058 ^abcd^	0.769 ± 0.051 ^abcd^	0.775 ± 0.048 ^abcdefg^	0.597 ± 0.037 ^cdefg^	202.5 ± 9.2 ^cd^
HS + SNP	0.679 ± 0.029 ^abcde^	0.819 ± 0.052 ^abcde^	0.758 ± 0.047 ^abcde^	0.769 ± 0.045 ^abcdefg^	0.606 ± 0.039 ^cdef^	198.3 ± 9.1 ^d^
HS + NaHS	0.674 ± 0.026 ^abcde^	0.811 ± 0.047 ^abcde^	0.749 ± 0.042 ^abcde^	0.754 ± 0.040 ^abcdefgh^	0.611 ± 0.041 ^cde^	191.4 ± 8.9 ^d^
HS + Eth + HT	0.538 ± 0.021 ^fgh^	0.760 ± 0.028 ^fgh^	0.676 ± 0.026 ^bcde^	0.624 ± 0.030 ^ghi^	0.667 ± 0.050 ^bc^	136.5 ± 8.1 ^efg^
HS + SNP + HT	0.529 ± 0.018 ^fgh^	0.751 ± 0.023 ^fgh^	0.654 ± 0.021 ^cde^	0.611 ± 0.027 ^hi^	0.679 ± 0.052 ^abc^	130.8 ± 7.4 ^fg^
HS + NaHS + NBD	0.576 ± 0.023 ^defgh^	0.780 ± 0.038 ^defgh^	0.705 ± 0.033 ^abcde^	0.656 ± 0.034 ^defghi^	0.635 ± 0.044 ^c^	146.6 ± 8.4 ^efg^
HS + NaHS + cPTIO	0.563 ± 0.022 ^efgh^	0.771 ± 0.030 ^efgh^	0.694 ± 0.030 ^abcde^	0.642 ± 0.032 ^efghi^	0.642 ± 0.046 ^c^	141.2 ± 8.2 ^efg^

**Table 4:** Hydrogen peroxide (H_2_O_2_), thiobarbituric acid reactive substances (TBARS) content, and activities of superoxide dismutase (SOD), ascorbate peroxidase (APX), and glutathione reductase (GR) in the leaves of rice (*Oryza sativa* L.) cultivar Taipei-309 after foliar treatment of plants with 200 µL L^−1^ ethephon (Eth), 100 µM sodium nitroprusside (SNP) or 200 µM sodium hydrosulfide (NaHS) grown with or without high temperature stress (HS; 40 °C) or 100 µM hypotaurine (HT), 100 µM 2-4-carboxyphenyl-4,4,5,5-tetramethylimidazoline-1-oxyl-3-oxide (cPTIO) or 100 µM norbornadiene (NBD) scavengers of hydrogen sulfide (H_2_S), nitric oxide (NO), and ethylene action inhibitors, respectively, with HS at 15 days after sowing. Data are presented as treatments mean ± SE (*n* = 4). The values followed by the same letters did not differ significantly by LSD test at *p* < 0.05. FW, fresh weight.

Treatments	H_2_O_2_ Content	TBARS Content	SOD Activity	APX Activity	GR Activity
	(nmol g^−1^ FW)	(U mg^−1^ Protein min^−1^)			
Control	28.3 ± 1.26 ^ghi^	10.6 ± 1.22 ^efghijk^	7.41 ± 0.43 ^mn^	1.35 ± 0.10 ^mn^	0.19 ± 0.01 ^jkl^
HS	86.7 ± 4.14 ^b^	22.1 ± 2.07 ^a^	11.0 ± 0.55 ^fghij^	1.94 ± 0.14 ^jklm^	0.27 ± 0.017 ^ghi^
Eth	21.1 ± 1.17 ^i^	7.1 ± 0.79 ^k^	13.9 ± 0.76 ^bcd^	2.97 ± 0.23 ^bcdef^	0.38 ± 0.028 ^bcd^
SNP	23.5 ± 1.20 ^hi^	7.9 ± 0.81 ^jk^	13.1 ± 0.62 ^cde^	2.73 ± 0.22 ^cdefgh^	0.36 ± 0.026 ^bcd^
NaHS	24.7 ± 1.22 ^ghi^	8.2 ± 0.97 ^ijk^	12.8 ± 0.50 ^cdef^	2.59 ± 0.20 ^efghi^	0.34 ± 0.025 ^cdef^
HS + Eth	29.4 ± 1.72 ^ghi^	10.9 ± 1.20 ^efghijk^	15.8 ± 0.98 ^a^	3.74 ± 0.29 ^a^	0.46 ± 0.034 ^a^
HS + SNP	30.2 ± 2.11 ^gh^	11.2 ± 1.25 ^efghijk^	14.9 ± 0.81 ^ab^	3.53 ± 0.26 ^ab^	0.42 ± 0.032 ^ab^
HS + NaHS	30.9 ± 2.51 ^gh^	11.5 ± 1.34 ^defghijk^	14.1 ± 0.79 ^bc^	3.32 ± 0.24 ^abc^	0.40 ± 0.030 ^abc^
HS + Eth + HT	61.3 ± 3.16 ^cde^	14.2 ± 1.55 ^bcdefg^	10.8 ± 0.49 ^ghij^	1.91 ± 0.16 ^jklm^	0.25 ± 0.015 ^hij^
HS + SNP + HT	64.1 ± 3.37 ^cd^	14.7 ± 1.65 ^bcdef^	9.7 ± 0.40 ^jkl^	1.87 ± 0.13 ^jklm^	0.24 ± 0.011 ^hijk^
HS + NaHS + NBD	51.8 ± 2.70 ^f^	13.3 ± 1.36 ^bcdefgh^	12.3 ± 0.65 ^cdefgh^	2.31 ± 0.19 ^ghijk^	0.29 ± 0.023 ^efgh^
HS + NaHS + cPTIO	53.2 ± 2.80 ^ef^	13.9 ± 1.49 ^bcdefg^	12.1 ± 0.58 ^defgh^	2.19 ± 0.18 ^hijkl^	0.28 ± 0.020 ^fgh^

**Table 5:** Hydrogen peroxide (H_2_O_2_), thiobarbituric acid reactive substances (TBARS) content, and activities of superoxide dismutase (SOD), ascorbate peroxidase (APX), and glutathione reductase (GR) in the leaves of rice (*Oryza sativa* L.) cultivar Rasi after foliar treatment of plants with 200 µL L^−1^ ethephon (Eth), 100 µM sodium nitroprusside (SNP) or 200 µM sodium hydrosulfide (NaHS) grown with or without high temperature stress (HS; 40 °C) or 100 µM hypotaurine (HT), 100 µM 2-4-carboxyphenyl-4,4,5,5-tetramethylimidazoline-1-oxyl-3-oxide (cPTIO), or 100 µM norbornadiene (NBD) scavengers of hydrogen sulfide (H_2_S), nitric oxide (NO), and ethylene action inhibitors, respectively, with HS at 15 days after sowing. Data are presented as treatments mean ± SE (*n* = 4). The values followed by the same letters did not differ significantly by LSD test at *p* < 0.05. FW, fresh weight.

Treatments	H_2_O_2_ Content	TBARS Content	SOD Activity	APX Activity	GR Activity
	(nmol g^−1^ FW)	(U mg^−1^ Protein min^−1^)			
Control	30.5 ± 1.85 ^gh^	12.2 ± 1.13 ^cdefghij^	6.38 ± 0.21 ^n^	1.24 ± 0.07 ^n^	0.16 ± 0.005 ^l^
HS	97.9 ± 5.46 ^a^	26.1 ± 2.11 ^a^	9.31 ± 0.34 ^jkl^	1.76 ± 0.12 ^klmn^	0.21 ± 0.011 ^ijkl^
Eth	24.6 ± 1.19 ^ghi^	8.5 ± 0.73 ^hijk^	11.7 ± 0.55 ^efghi^	2.67 ± 0.19 ^defghi^	0.29 ± 0.017 ^efgh^
SNP	26.9 ± 1.24 ^ghi^	9.7 ± 0.87 ^ghijk^	10.9 ± 0.51 ^ghij^	2.44 ± 0.17 ^fghij^	0.28 ± 0.015 ^fgh^
NaHS	28.0 ± 1.32 ^ghi^	10.0 ± 1.02 ^fghijk^	10.5 ± 0.45 ^hijk^	2.32 ± 0.16 ^ghijk^	0.26 ± 0.011 ^hi^
HS + Eth	31.8 ± 2.46 ^gh^	12.8 ± 1.22 ^bcdefghi^	13.2 ± 0.66 ^bcde^	3.27 ± 0.24 ^abcd^	0.38± 0.026 ^bcd^
HS + SNP	32.9 ± 2.60 ^g^	13.3 ± 1.30 ^bcdefgh^	12.6 ± 0.61 ^cdefg^	3.10 ± 0.21 ^bcde^	0.35 ± 0.023 ^cde^
HS + NaHS	33.2 ± 2.88 ^g^	13.8 ± 1.53 ^bcdefg^	11.9 ± 0.57 ^efghi^	2.90 ± 0.20 ^cdefg^	0.33 ± 0.020 ^defg^
HS + Eth + HT	65.8 ± 3.56 ^cd^	16.9 ± 1.92 ^bc^	8.8 ± 0.30 ^klm^	1.71 ± 0.13 ^klmn^	0.19 ± 0.01 ^jkl^
HS + SNP + HT	69.7 ± 4.05 ^c^	17.3 ± 2.04 ^b^	8.0 ± 0.25 ^lmn^	1.67 ± 0.11 ^lmn^	0.18 ± 0.01 ^kl^
HS + NaHS + NBD	57.4 ± 2.90 ^def^	15.4 ± 1.72 ^bcde^	10.1 ± 0.40 ^ijk^	2.05 ± 0.18 ^ijkl^	0.24 ± 0.015 ^hijk^
HS + NaHS + cPTIO	59.3 ± 3.17 ^def^	16.2 ± 1.81 ^bcd^	9.7 ± 0.35 ^jkl^	1.96 ± 0.16 ^jklm^	0.23 ± 0.011 ^hijk^

## Data Availability

The data presented in this study and the Supplementary Materials are available in the graphs provided in the manuscript.

## Supplementary Materials

The following are available online at https://www.mdpi.com/article/10.3390/antiox11081478/s1, File S1: Material and methods details, Table S1: Primer pairs used for quantitative RT-PCR.
