# Supplementary material for: Hydrogen Sulfide, Ethylene, and Nitric Oxide Regulate Redox Homeostasis and Protect Photosynthetic Metabolism under High Temperature Stress in Rice Plants
Source: Antioxidants (Basel). 2022 Jul 28;11(8):1478. doi: 10.3390/antiox11081478 (PMC9405544; doi:10.3390/antiox11081478)
Supplement: Supplementary file 1 [file antioxidants-11-01478-s001.zip › antioxidants-1792564-supplementary/Supplementary File S1.pdf]

Supplementary File S1: Methodology and Table S1

Hydrogen Sulfide, Ethylene and Nitric Oxide Regulate Redox Homeostasis and Protect Photosynthetic Metabolism Under High Temperature Stress in Rice Plants

Harsha Gautam [1], Mehar Fatma[1], Zebus Sehar [1], Iqbal R. Mir[1] and Nafees A. Khan [1,*]

[1]Plant Physiology and Biochemistry Laboratory, Department of Botany, Aligarh Muslim University, Aligarh, 202002, India; harshagautam99@gmail.com (H.S.); meharfatma30@gmail.com (M.F.); seharzebus5779@gmail.com (Z.S.); m3riqbal@gmail.com (I.R.M.)
[1]*Correspondence: naf9.amu@gmail.com (N.A.K.)

Chlorophyll Fluorescence Measurement

Fully expanded leaves were allowed to adapt under dark for 30 min before chlorophyll fluorescence measurements using Junior-PAM chlorophyll fluorometer (Heinz Walz, Germany) were made. Actual pigment system (PS) II, maximal efficiency of PSII, intrinsic PSII efficiency, photochemical quenching, non-photochemical quenching and electron transport rate were calculated. The details of the procedure are given in Supplementary File S1. Minimal fluorescence (Fo) and maximum fluorescence (Fm) were measured in dark-adapted leaves with a low measuring beam at a light intensity of 125 μmol m^{-2} s^{-1}, whereas under light-adapted conditions, minimal fluorescence (Fo′) and maximum fluorescence (Fm′) were measured in the same leaves with saturating light intensity (720 μmol m^{-2} s^{-1}) together with steady-state fluorescence (Fs). The variable fluorescence (Fv and Fv′) was calculated using the values of Fm-Fo and Fm′-Fo′, and actual PSII efficiency (Φ PSII) was determined as Fm′-Fs/Fm′, maximal efficiency of PSII by using Fv/Fm and intrinsic PSII efficiency of PSII by using Fv′/Fm′. Using fluorescence parameters determined in both the light- and dark-adapted states, the photochemical quenching (qP) and non-photochemical quenching (NPQ) parameters were calculated. Photochemical quenching was calculated as (Fm′-Fs)/Fv′ and NPQ as (Fm-Fm′)/Fm′ (Maxwell and Johnson, 2000). Electron transport rate (ETR) was calculated by the following formula: Φ PSII × photosynthetic photon flux density × 0.5 × 0.84 as suggested by Krall and Edwards (1992).

Measurement of Hydrogen Peroxide and Lipid Peroxidation

The assay of H_2O_2 was done following the method of Okuda et al. (1991). Fresh leaf tissues (500 mg) were homogenized in ice-cold 200 mM perchloric acid ($HClO_4$), followed by centrifugation at 1200 × g for 10 min. Later the supernatant was neutralized with 4 M KOH. Homogenate was further centrifuged at 500 × g for 3 min for the removal of insoluble potassium perchlorate. The reaction mixture (1.5 mL) contained 1

mL of the eluate, 80 μL of 3-methyl-2-benzothiazoline hydrazone, 400 μL of 12.5 mM 3-(dimethylamino) benzoic acid in 0.375 M phosphate buffer (pH 6.5), and 20 μL of peroxidase (0.25 unit). The reaction was initiated with the addition of peroxidase at 25°C. The increase in absorbance was estimated at 590 nm on a spectrophotometer.

Lipid peroxidation was estimated by measuring the content of thiobarbituric acid reactive substance (TBARS) as described by Dhindsa et al. (1981). Fresh leaf samples (500 mg) were ground in 0.25% 2-thiobarbituric acid (TBA) in 10% trichloroacetic acid (TCA) using mortar and pestle. The mixture was heated at 95°C for 30 min and rapidly cooled on ice bath. Then, mixture was allowed to centrifuge at 10,000 × g for 10 min. To one mL of the supernatant 4.0 mL of 20% TCA containing 5% TBA was added. The absorbance of the supernatant was read at 532 nm and corrected for non-specific turbidity by subtracting the absorbance of the same at 600 nm. The content of TBARS was calculated using the extinction coefficient (155 mM^{-1} cm^{-1}).

Assay of Antioxidant Enzymes

Fresh leaf tissue (200 mg) was collected from the third leaves and ground rapidly in ice-cold extraction buffer containing potassium-phosphate buffer (100 mM, pH 7.0), 0.05% (v/v) Triton X-100 and 1% (w/v) PVP. The homogenates were centrifuged at 15,000×g for 20 min at 4°C. The clear supernatant obtained after centrifugation was used for the assay of enzymes. For the assay of ascorbate peroxidase (APX), the extraction buffer was supplemented with 2 mM ascorbate. Protein was estimated according to the protocol described by Bradford (1976), utilizing Bovine serum albumin as a protein standard.

Superoxide dismutase (SOD) activity was determined according to Beyer and Fridovich (1987) and Giannopolitis and Ries (1977) which is based on inhibition of the photochemical reduction of nitro blue tetrazolium (NBT). Five mL of reaction mixture containing 5.0 mM HEPES (pH 7.6), 0.1 mM EDTA, 50 mM Na_2CO_3 (pH 10.0), 13 mM methionine, 0.025% (v/v) Triton X-100, 63 μmol NBT, 1.3 μmol riboflavin and the enzyme extract was illuminated for 15 min (360 μmol m^{-2} s^{-1}). A control set was also illuminated to correct for background absorbance. A unit of SOD was defined as the amount of enzyme that inhibited the NBT reduction by 50% at 560 nm.

The ascorbate peroxidase (APX) activity was determined according to the method of Nakano and Asada (1981). The assay mixture (1.0 mL) contained phosphate buffer (50 mM, pH 7.0), 0.1 mM EDTA, 0.5 mM ascorbate, 0.1 mM H_2O_2, and enzyme extract. Final volume was observed at 290 nm for 1 min using spectrophotometer. A decrease in absorbance was observed as soon as the reaction was started by adding

H_2O_2. APX activity was calculated with the extinction coefficient of 2.8 mM^{-1} cm^{-1}. One unit of enzyme is the amount necessary to decompose 1 μmol of substrate per min at 25°C.

Glutathione reductase (GR) activity was determined following the method of Foyer and Halliwell (1976) by monitoring the glutathione (GSH) dependent oxidation of nicotinamide adenine dinucleotide phosphate (NADPH) at 340 nm. The reaction mixture (3.0 mL) contained phosphate buffer (25 mM, pH 7.8), 0.5 mM GSSG, 0.2 mM NADPH, and the enzyme extract. The reaction started with the addition of GSSG, and the absorbance showed a decreasing trend. The activity of GR was calculated by using the extinction coefficient of (6.2 mM^{-1} cm^{-1}). One unit of enzyme is the amount necessary to decompose 1 μmol of NADPH min^{-1} at 25°C.

Ethylene Evolution

The evolution of ethylene in leaves was measured using a gas chromatograph, as described previously by Fatma et al. (2021a). The details are given in Supplementary File S1. Leaf samples were cut into small pieces and placed in 30 mL tubes containing moist paper to prevent evaporation from the tissue, which were then sealed with secure rubber caps and exposed to light for 2 h under the same conditions as for plant growth. With a hypodermic syringe, a 1 mL gas sample was taken from the tubes and analyzed on a gas chromatograph (Nucon 5700, New Delhi, India) equipped with a 1.8 m Porapack N (80–100 mesh) column, a flame ionization detector, and a data station. As a carrier gas, nitrogen was used. Nitrogen, hydrogen, and oxygen flow rates were 30, 30, and 300 mL min^{-1}, respectively. The temperature of the detector was set at 150°C. Based on the retention time, ethylene was detected and measured by comparing peaks from standard ethylene concentrations.

RNA Isolation and cDNA Synthesis

Total RNA was isolated from rice leaves using TRIzol reagent (Ambion, Life Technologies, USA) according to the manufacturer's instructions. With the help of a Nanodrop spectrophotometer (Thermo Scientific, USA), the extracted RNA was quantified. To ensure the integrity of the RNA, each sample was run on agarose formaldehyde gel Turano et al. (1997). The first-strand of the cDNA was made from 1 μg of total RNA of control and treated samples. The cDNA template was synthesized using the reaction mixture containing 20 U/μL Moloney murine leukemia virus reverse transcriptase (MuMLV) enzyme (Fermentas, USA) at 42°C for 50 min and at 70°C for 10 min. The reverse transcription reaction was carried out using 2.5 μM Oligo (dT) 18 primer (Fermentas, USA) and 10 mM dNTPs. Primers for gene

expression analysis were designed using online primer designing software (IDT) and cDNA sequences of selected genes were obtained from NCBI.

Quantitative Real-Time PCR Analysis

Real-time PCR (RT-PCR) was performed in 96-well reaction plate (Roche, Germany) containing 20 μL reaction mixture of × 10 reaction buffer, 2 mM dNTPs, 1 mM $MgCl_2$, 0.35 μM each of forward and reverse primers, 1 μL Sybr green (×10), 10 μg cDNA template and 5 U Taq polymerase on a thermal cycler (Light cycler 480 II, Roche, Germany). All quantifications were normalized to actin DNA fragment amplified by β-actin forward and β-actin reverse primers. The actin gene was used as an internal control for evaluating the efficiency of RT-PCR for particular genes. PCR cycling conditions were as follows: denaturation at 95°C for 3 min, 40 cycles of 95°C (20 s), 66°C (1 min) and 72°C (1 min) with 5 min of final extension at 72°C. The amplified product was resolved on 1.2% agarose gel. The specificity of amplicons was verified by melting curve analysis (60 to 95°C) after 40 cycles. All reactions were performed in three biological replicates (with three technical replicates of each), using gene-specific primers and actin primers as an internal control. The data were taken as the expression of the gene of interest in relation to the internal control in the treated sample compared with the untreated control.

References

Beyer Jr, W.F.; Fridovich, I. Assaying for superoxide dismutase activity: some large consequences of minor changes in conditions. *Anal. Biochem.* **1987,** *161*, 559-566.

Bradford, M.M. A rapid and sensitive method for the quantitation of microgram quantities of protein utilizing the principle of protein-dye binding. *Anal. Biochem.* **1976**, *72*, 248-254.

Dhindsa, R.S.; Plumb-Dhindsa, P.A.M.E.L.A.; Thorpe, T.A. Leaf senescence: correlated with increased levels of membrane permeability and lipid peroxidation, and decreased levels of superoxide dismutase and catalase. *J. Exp. Bot.* **1981**, *32*, 93-101.

Fatma, M.; Iqbal, N.; Gautam, H.; Sehar, Z.; Sofo, A.; D'Ippolito, I.; Khan, N.A. Ethylene and sulfur coordinately modulate the antioxidant system and ABA accumulation in mustard plants under salt stress. *Plants* **2021**, *10*, 180.

Foyer, C.H.; Halliwell, B. The presence of glutathione and glutathione reductase in chloroplasts: a proposed role in ascorbic acid metabolism. *Planta* **1976**, *133*, 21-25.

Giannopolitis, C.N.; Ries, S.K. Superoxide dismutases: I. Occurrence in higher plants. *Plant Physiol.* **1977**, *59*, 309-314.

Krall, J.P.; Edwards, G.E. Relationship between photosystem II activity and CO_2 fixation in leaves. *Physiol. Plantar.* **1992**, *86*, 180-187.

Maxwell, K.; Johnson, G.N. Chlorophyll fluorescence—a practical guide. *J. Exp. Bot*. **2000**, *51*, 659-668.

Nakano, Y.; Asada, K. Hydrogen peroxide is scavenged by ascorbate-specific peroxidase in spinach chloroplasts. *Plant Cell Physiol.* **1981**, *22*, 867-880.

Okuda, T.; Matsuda, Y.; Yamanaka, A.; Sagisaka, S. Abrupt increase in the level of hydrogen peroxide in leaves of winter wheat is caused by cold treatment. *Plant Physiol.* **1991**, *97*, 1265-1267.

Turano, F.J.; Thakkar, S.S.; Fang, T.; Weisemann, J.M. Characterization and expression of NAD (H)-dependent glutamate dehydrogenase genes in Arabidopsis. *Plant Physiol.* **1997**, *113*, 1329-1341.

Table S1. Primer pairs used for quantitative RT-PCR

S.No.	Gene	Forward primer	Reverse primer
1	*psbA*	ATATTGTGGCCGCTCAT	TCCGTTTAGATTGAAAGCCATAG
2	*psbB*	GCCGGAACTATGTGGTATG	GACCAAGCTTCTGATAAACTGAG
3	*Mn-SOD* (manganese-superoxide dismutase)	CAAGGATCTGGATGGGTG	CAGGTAGTACGCATGCTC
4	*Fe-SOD* (iron-superoxide dismutase)	TATACGCTCAGCATTATCACTTT	AACATACATTCGCCTGTCA
5	*Cu-SOD*	GCTAATATCCATGTTGTTGACAG	GAAGTCCGATGATCCCG
6	*APX* (thylakoid-bound ascorbate peroxidase)	TGATCATCTTAGGGAGGTATTCTACA	TGATTGCCCTCCAGGTTC
**Reference gene primer sequences used for quantitative RT-PCR**			
1	Actin	GACTGCCAAGACCAGCTCC	CTTCCTAATATCCACGTCGCAC
